# Next-Generation SERS Probes: Engineering Hotspots, Intelligent Molecular Targeting, and AI-Driven Spectral Analysis for Emerging Applications

**DOI:** 10.3390/nano16100628

**Published:** 2026-05-19

**Authors:** Unmanaa Dewanjee, Shi Bai, Yury V. Ryabchikov, David Fieser, Sharma Pradakshina, Jie Jayne Wu, Marco Fronzi, Anming Hu

**Affiliations:** 1 Department of Mechanical and Aerospace Engineering, University of Tennessee Knoxville, 1512 Middle Drive, Knoxville, TN 37996, USA; 2 Advanced Laser Processing Research Team, RIKEN Center for Advanced Photonics, 2-1 Hirosawa, Wako 351-0198, Saitama, Japan; 3 FZU—Institute of Physics of the Czech Academy of Sciences, Na Slovance 1999/2, 182 00 Prague, Czech Republic; 4 Department of Electrical Engineering and Computer Science, University of Tennessee Knoxville, 1512 Middle Drive, Knoxville, TN 37996, USA; 5 School of Physics, The University of Sydney, Sydney, NSW 2006, Australia

**Keywords:** SERS, plasmonic hotspots, large-area nanomanufacturing, molecular sensing, density function theory, machine learning

## Abstract

Surface-enhanced Raman spectroscopy (SERS) has evolved from a fundamental optical phenomenon to a powerful, molecule-specific analytical technique capable of detecting ultra-trace-level species across biomedicine, catalysis, environmental monitoring, and national security applications. In this review, we summarize recent advances in SERS probe design and fabrication along three major directions: (i) engineering plasmonic hotspots with enhanced field confinement to achieve stronger and more uniform signals; (ii) analyte-directed strategies that precisely position and retain target molecules via tailored surface chemistries, nanoscale confinement, and on-surface reactions for single hotspot SERS; and (iii) hybrid architectures integrating plasmonic metals with functional materials, including high entropy materials, semiconductors, and graphene and other 2D materials, to synergistically couple electromagnetic and chemical enhancement mechanisms. Despite significant progress, key challenges remain for practical applications outside laboratories, including substrate reproducibility and stability, diverse analyte compatibility, unknown molecule identification and standardized quantitative performance in complex environments. We highlight emerging solutions, such as large-area nanomanufacturing for controlled nanoscale gaps, high-resolution Raman mapping for spatial–temporal characterization, density-functional-theory-guided molecular interpretation, and machine-learning-enabled spectral analysis. Advances in foundational AI models and data-driven discovery are positioning SERS to become an increasingly versatile platform, from decoding unknown molecular structures to analyzing complicated multi-component systems for environmental, biomedical, and national security applications with high sensitivity and selectivity.

## 1. Introduction: Fundamentals of SERS Enhancement

Surface-enhanced Raman spectroscopy (SERS) is an advanced vibrational spectroscopic technique in which the inherently weak Raman scattering from molecules is amplified, often by many orders of magnitude [1,2,3,4]. This occurs when the target analyte is within a few nanometers of nanostructured enhancing surfaces, most commonly plasmonic Ag or Au nanostructures [2,5,6,7]. This enhancement arises primarily from the strong localization of electromagnetic fields into nanoscale regions known as hotspots, and, in some cases, from molecule–surface interactions that modify the effective Raman polarizability [8,9,10]. The significance of SERS is that it retains Raman’s molecular “fingerprint” specificity while overcoming Raman’s sensitivity limitation, enabling trace-level and even single- or few-molecule detection under favorable conditions, thereby making it attractive for rapid chemical identification in realistic, complex samples [3,11,12]. Consequently, SERS has emerged as a powerful platform for ultrasensitive sensing and analysis across diverse fields, including biomedical diagnostics [13], environmental monitoring [14,15], food safety [16], and astrophysics [17,18], where small sample volumes, fast readout, high chemical specificity, and remote sensing are essential.

The origins of SERS trace back to 1974, when Martin Fleischmann and his colleagues measured pyridine on a rough silver electrode with a Raman spectrometer and found its Raman intensity was hundreds of times higher than typical results. They attributed the unusual Raman signals to the roughness of the silver electrode because the amount of pyridine molecules might be increased on a rough surface, leading to the improvement of Raman signals [19]. Two years later, in 1977, two independent groups found that electromagnetic effects associated with surface roughness and localized plasmon excitation contributed to the enhanced Raman signals [20,21]. This led to the formal recognition of surface-enhanced Raman scattering with an additional chemical enhancement mechanism [22,23] in the 1980s. A major milestone in the evolution of SERS was the single molecule detection employed in the 1990s, which had been theoretically predicted using noble metal nanostructures and was subsequently achieved experimentally [11,24]. From 2000, research has increasingly focused on the development of optimal SERS probes, including studies of material modification and geometry design to achieve the highest Raman signals [25,26]. Today, SERS remains a rich resource for scientific discovery. It has found widespread applications across numerous disciplines, and its impact continues to grow at the cutting edge of advanced materials, analytical technologies, and precision medicine.

Despite the remarkable sensitivity and molecular specificity of SERS, practical and reproducible SERS sensing remains a significant challenge [27,28]. SERS signals are often dominated by a small number of nanoscale “hotspots,” so its performance can vary strongly with substrate morphology [4,10], nanogap statistics [29], and surface chemistry [30]. This variability complicates quantitative analysis and comparisons across studies. This article consolidates progress in SERS probe design and highlights common principles for developing substrates that are not only highly enhancing, but also reproducible, stable, and suitable for application-oriented sensing. Although there have been several comprehensive reviews [31,32,33], this study highlights the development of robust SERS probes for various emergent applications with integrated computational spectra analysis and machine learning algorithms.

**Figure 1:**
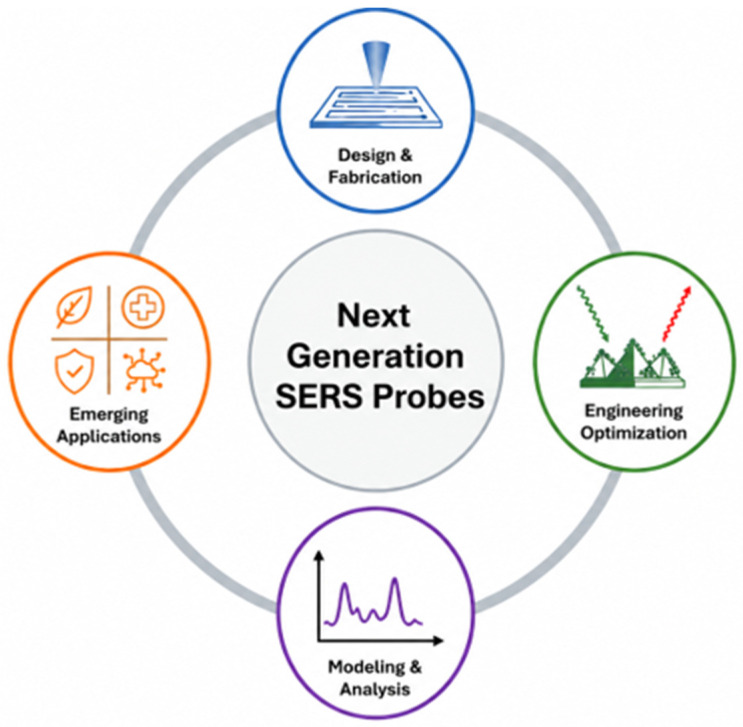
Schematic overview of the key components of next-generation SERS probe development.

This review is organized as shown in Figure 1. We begin with a discussion of the fundamental enhancement mechanisms of SERS. Chapter 2 provides an overview of the fabrication and microstructure control of SERS substrates. In Chapter 3, we discuss strategies for optimizing SERS probes, followed by a chapter reviewing density functional theory (DFT) calculations and machine-learning approaches for SERS analysis and unknown molecule identification. Subsequently, we highlight emerging applications of SERS for environmental monitoring, biomedical diagnostics, and national security sensing. Finally, we conclude this review by outlining the remaining challenges and possible research directions in the near future.

### 1.1. Electromagnetic vs. Chemical Enhancement

**Figure 2:**
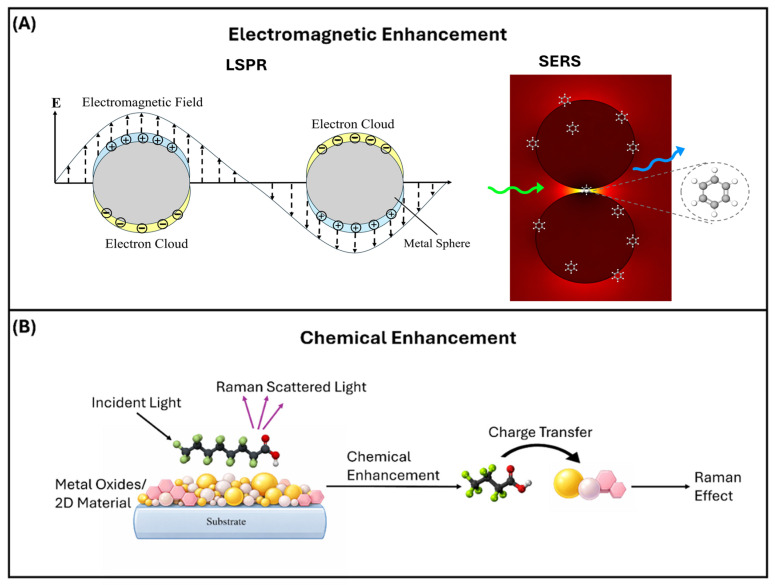
Schematic diagram for (**A**) electromagnetic enhancement and (**B**) chemical enhancement for SERS.

There are typically two enhancement pathways related to SERS. The first is called electromagnetic (EM) enhancement, which occurs through a locally amplified field mediated by the surface plasmons present on the nanostructured metallic substrate created for SERS use [34]. The second pathway involves the action or bonding between the adsorbate and the SERS substrate, which we often refer to as charge transfer-assisted Raman processes [35]. Figure 2 shows the schematic of these two enhancement mechanisms for SERS [3,36].

Electromagnetic SERS enhancement is commonly described as a two-step process in which the Raman response is amplified through (i) local-field enhancement through excitation and (ii) radiation (emission) enhancement during scattering [37,38,39,40,41]. The Raman process itself consists of an excitation step (induced dipole formation) and an emission step (radiation from the Raman dipole), and under the weak-coupling approximation the enhancement can be treated as the product of two field-enhancement factors [42]. In near plasmonic metals, such as, Ag, Au, Cu [43,44], the local-mode density is increased, providing many additional pathways for both excitation and emission relative to free space; this is generally identified as the physical origin of EM enhancement in SERS [42]. Consequently, the total EM enhancement factor for a single molecule (SMEF) is written as the product of an excitation local-field factor and a radiation local-field factor:

(1) $$SMEF\approx \left|M_{1}{\left(\omega_{L}\right)}^{2}\right|\left|M_{2}{\left(\omega_{R}\right)}^{2}\right|$$

where $M_{1}$ and $M_{2}$ quantify the plasmon-mediated enhancement associated with the coupling of the plasmon resonance to the incident (laser) and Raman-scattered light, respectively. Under conditions where optical reciprocity applies, both steps can often be treated as being generated by a common plasmon mode (i.e., $M_{1}\approx M_{2}$), leading to the widely used field-ratio expression [42] below:

(2) $${\left|M_{Total}\right|}^{2}={\left|\frac{E_{loc}\left(\omega_{L}\right)}{E_{I}\left(\omega_{L}\right)}\right|}^{2}{\left|\frac{E_{loc}\left(\omega_{R}\right)}{E_{I}\left(\omega_{R}\right)}\right|}^{2}$$

This two-step formulation is particularly useful because it makes explicit that strong SERS requires not only intense excitation near-fields, but also efficient plasmon-mediated out-coupling of the Raman dipole to the far field [42]. The spectral dependence of $|M_{1}(\omega ){|}^{2}$ is expected to resemble the plasmon resonance spectrum, since the radiation enhancement is mediated by plasmonic reradiation of the Raman dipole; accordingly, validating the two-fold mechanism can be approached by comparing SERS spectral envelopes with plasmon resonance (elastic scattering/extinction) spectra in well-defined hotspot geometries such as nanoparticle dimers [42].

Since electromagnetic (EM) SERS enhancement mainly comes from plasmonic near-fields, nanostructured metals (typically Ag/Au/Cu) act like optical antennas that concentrate the laser field near the surface, so the Raman signal increases strongly when the plasmon resonance is well matched to the excitation/scattered wavelengths [2,20,34,42,45]. In practice, the plasmon resonance is controlled by particle size or shape, sharpness, and the surrounding medium/coatings [46,47,48,49], and better overall enhancement is often achieved when the plasmon peak is tuned slightly red of the laser to account for the Raman Stokes shift [32]. The strongest EM gains usually come from hotspots, especially nanogaps or junctions (e.g., dimers or particle–film cavities) [29,31,45,50], where coupling creates extremely confined fields. Engineered substrates can additionally use collective resonances (arrays, cavity-like modes) and polarization control to access stronger or more robust hotspot excitation [51,52,53].

However, simply shrinking gaps has limits. When gaps approach the sub-nanometer regime, tunneling/nonclassical effects can reduce the classical field growth expected from ever-smaller gaps [32,54,55]. Because EM enhancement is highly nonuniform, measured signals are often dominated by a few hotspots; therefore, reported enhancement factors depend strongly on how “active molecules” are counted and should be supported by mapping/statistics and careful EF methodology [12,31,56].

Chemical enhancement (chemical mechanism, CM) is attributed to adsorption-driven changes in the electronic structure of the molecule–surface complex that modify the Raman polarizability, and the measured SERS intensity is commonly treated as the product of EM and CM contributions rather than an either/or mechanism [32,57,58]. CM is often grouped into (i) resonance Raman, (ii) substrate–molecule charge-transfer (CT) resonances, and (iii) adsorption-induced polarizability changes, and CM tends to become more apparent when excitation is off the LSPR or when CT transitions are brought into resonance [32,57,59,60].

A key experimental hallmark is that CM is short-range/“first-layer”-dominated because it requires strong electronic coupling (wavefunction overlap) between the adsorbate and surface, so chemisorbed molecules in direct contact can exhibit larger cross sections than molecules farther from the interface [59,61]. Microscopically, CM is frequently rationalized by chemisorption-induced hybridization, where new metal–molecule (or substrate–molecule) states act as intermediate resonant states for Raman scattering, enabling CT excitations at lower energies than the free-molecule electronic transitions when the substrate Fermi level (or band edges) lies appropriately relative to the molecular HOMO/LUMO [60,62,63,64].

Because CM depends on the details of the interface, it is highly sensitive to the adsorption site or to the orientation and local environment, and modern perspectives emphasize that while EM provides most of the magnitude, CM largely drives spectral changes and must be treated explicitly, especially when interpreting chemical information from SERS [57,58,59]. A particularly direct method for probing CT is electrochemical tuning; changing potential shifts interfacial level alignment (and local fields), producing potential-dependent relative intensities and band shifts that are often interpreted through CT resonance conditions, while also requiring care because reorientation and Stark effects can contribute [58,59,65].

On semiconductors and 2D materials, where visible–NIR LSPR is typically weak or absent, CM/CT pathways are often the dominant source of enhancement; this is commonly framed in terms of donor (HOMO/VB) to acceptor (LUMO/CB) alignment plus intensity borrowing, and some systems show strong asymmetry where VB-to-LUMO (“C-term”) CT can be far more efficient than HOMO-to-CB (“B-term”) CT [32,60,66,67].

Current modeling work highlights that CM cannot be separated from real experimental complexity; solvents and ligands can reshape near-field distributions and even contribute effects comparable to CM, while halides can alter aggregation/hotspot formation and binding affinity, and thereby modulate EM, CM, and CT resonances 50 [57,58,59].

The SERS enhancement factor is defined as [68,69,70] follows:

(3) $$EF=\frac{I_{SERS}/N_{ads}}{I_{RAMAN}/N_{bulk}}$$

where *I_SERS_* and *I_RAMAN_* are the Raman intensities measured with and without plasmonic enhancement, and *N_ads_* and *N_bulk_* are the number of molecules contributing to each signal [68,69,70]. Based on EM enhancement, the typical EF is usually on the order of $10^{3}$–$10^{12}$, while this EF for a CM/CT mechanism is in the range of 10–$10^{7}$ [58,71].

### 1.2. Spatial vs. Temporal Resolution

SERS measurements are inherently spatiotemporal because enhancement is dynamically concentrated at the vicinity of nanoscale hotspots. Therefore, the measured spectrum depends on both the measurement location (which hotspots are sampled) and the measurement time (how hotspot occupancy and hotspot structure evolve). This becomes especially important when the goal is reproducible sensing or tracking dynamic chemical/biological processes, where both hotspot distribution and hotspot stability shape the observed spectra [72,73]. The strong sensitivity of SERS signals to spatial and temporal variations originates from the electromagnetic scaling of Raman enhancement near plasmonic nanostructures. Especially in the electromagnetic mechanism, the Raman intensity is approximately proportional to the fourth power of the local electric field amplitude. As a consequence, even small variations in nanogap geometry, particle coupling, or molecular position within a hotspot can produce orders-of-magnitude changes in the measured signal. This extreme field sensitivity explains why SERS signals are often dominated by a small number of hotspots and why both spatial heterogeneity and temporal fluctuations are intrinsic features of SERS measurements.

Spatial resolution in SERS refers to how well one can distinguish where the signal originates across a substrate or within a sample. In practice, the strongest SERS signals often come from a small subset of hotspots, so spatial maps commonly show highly nonuniform intensity distributions even on nominally “uniform” substrates. This heterogeneity is widely recognized as a major source of variability and is one reason single-point “best spectrum” reporting can be misleading for comparing substrates or making quantitative claims [73,74]. An interesting phenomenon is anisotropic enhancement by oriented nanowire arrays [75], where the SERS signal displays rotational periodicity. With highly ordered nanophotonic structures, a Fano-coupling enhancement is remarkable [76]. To address spatial heterogeneity, modern SERS studies increasingly rely on mapping and statistics (e.g., intensity distributions and variability metrics) rather than isolated spectra. This mapping-based reporting is particularly important for real samples, where analyte distribution, transport limitations, and nonspecific adsorption can further confound the interpretation of measurements at a single spot [73,77].

Temporal resolution describes the ability to capture time-dependent changes in SERS intensity and spectral features. Time variations are frequently observed as “blinking” or intermittent bursts and can arise from multiple coupled factors, including analyte adsorption/desorption and reorientation, changes in the local dielectric environment, and nanoscale hotspot restructuring under illumination or in liquid environments. These fluctuations are not merely noise: they can encode mechanistic information about interfacial dynamics and rare molecular events [73,78,79].

A key limitation of conventional (long integration) SERS is that time averaging can obscure transient states and bias spectra toward rare high-intensity bursts. High-speed measurements directly demonstrate that a nanoparticle can remain “dark” most of the time and then produce short-lived bright events, meaning that temporal sampling strongly influences what is interpreted as the “representative” spectrum [73,78].

Dynamic SERS is built around short acquisition windows (typically milliseconds down to microseconds) and repeated sampling, which allow time-dependent events to be captured without being averaged out. A foundational concept is that high-frequency acquisition combined with statistical treatment can help separate true SERS contributions from background or normal Raman signals, particularly in challenging environments such as strongly scattering liquids [80].

**Figure 3:**
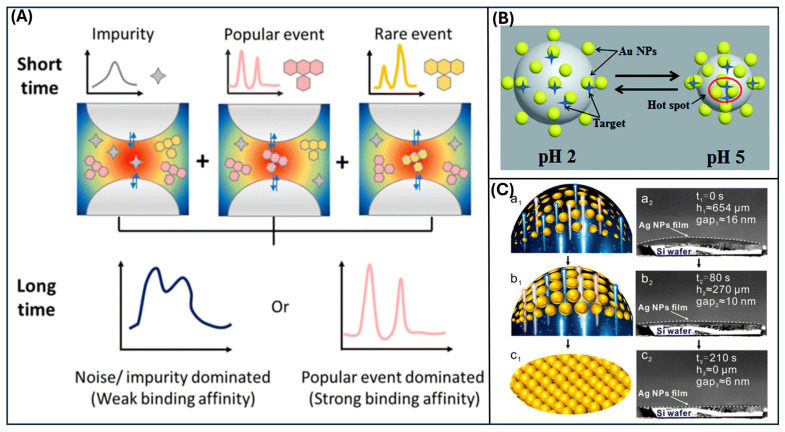
(**A**) Schematic comparison of conventional long-exposure SERS and dynamic short-exposure SERS for distinguishing noise, common events, and rare molecular events. Reproduced with permission from ref. [73]. Copyright 2025 Royal Society of Chemistry. (**B**) Schematic of pH-responsive AuNP/P2VP microgels for dynamic SERS hotspot formation. Reproduced with permission from ref. [84]. Copyright 2017 Royal Society of Chemistry. (**C**) AgNPs-decorated Basil-seeds microgels (ABM) platform for dynamic SERS. Reproduced with permission from ref. [85]. Copyright 2021 Journal of the American Chemical Society.

Dynamic SERS is also particularly valuable for capturing rare or weakly bound interactions. For example, dynamic SERS has been used to reveal single-molecule-level rare events that are difficult to observe when dwell times at hotspots are short and conventional spectra are dominated by background or intermittency. This difference between long-time and short-time SERS acquisition is schematically shown in Figure 3A, where dynamic SERS helps distinguish rare molecular events that may be hidden in conventional averaged spectra [73,79].

Studies in recent years have pushed dynamic SERS toward microsecond-resolved observation of SERS fluctuations and hotspot intermittency. High-speed imaging of SERS has shown that fluctuations can occur on very short time scales and can be spatially localized on individual nanoparticles, reinforcing the view that nanoscale hotspot dynamics (not only molecular diffusion) can dominate temporal behavior [78].

A parallel trend is the emergence of active control of hotspot occupancy to improve temporal stability and interpretability. Electro-plasmonic trapping in nanoholes, for instance, has been used to increase molecular residence time in hotspots to minutes, enabling the discrimination of single DNA bases and highlighting how controlling occupancy directly links temporal resolution with chemical specificity [81].

Similarly, plasmonic optical trapping—“plasmonic tweezers”—approaches build dynamic nanocavities that improve field reproducibility and enable high-throughput single-molecule SERS characterization in aqueous environments [82,83].

Recent probe designs explicitly target spatiotemporal reliability by integrating material function with hotspot engineering. Stimulus-responsive plasmonic microgels have been used to dynamically tune hotspot configurations and implement suction–release strategies for time-dependent detection in complex matrices, illustrating how engineered dynamics can be leveraged rather than avoided. A representative example is shown in Figure 3B, where pH-responsive AuNP/P2VP microgels shrink from pH 2 to pH 5, narrowing the interparticle gaps and generating dynamic SERS hotspots [84].

In wearable sensing, a hydrogel-based SERS chip with plasmonic trimers has been demonstrated for noninvasive biomarker monitoring (uric acid in sweat), showing how soft materials can improve analyte transport to hotspots and enable practical time-relevant readouts [74].

In intracellular monitoring, reversible SERS nanosensors have been developed to track redox dynamics in living cells, demonstrating time-dependent SERS capabilities in chemically complex environments where stability and anti-interference are essential [86].

Complementary efforts, such as SERS endoscopy using plasmonic nanowire probes, enable the monitoring of intracellular drug fate/dynamics, illustrating a growing emphasis on time-resolved SERS readouts in biomedical contexts [77]. Another way to improve sensitivity is to move analyte molecules into the hotspot regions, such as a nanocapillary pumping system that actively drives target molecules into the hotspots between nanoparticles. Figure 3C shows a schematic of the early shrinking stages of AgNP-decorated basil-seed microgels (ABM), along with the corresponding SEM images [73].

Beyond point measurements and 2D mapping, SERS is increasingly used for 2D and 3D imaging to localize chemical information across heterogeneous biological and environmental samples. In 2D imaging, SERS maps provide spatially resolved distributions of target species, which often use SERS tags for multiplexing; however, quantitative interpretation still requires careful handling of hotspot heterogeneity, probe distribution, and background interference [87]. Extending to 3D, confocal *z*-stack acquisition and 3D-model systems (e.g., spheroids and engineered scaffolds) enable volumetric SERS readouts that better reflect in-vivo-like transport and microenvironment effects, and recent studies have demonstrated proof-of-concept 3D SERS imaging in dense 3D cell-culture models and SERS-enabled 3D scaffolds for monitoring tumor evolution and spatial biochemical gradients over time [88,89]. More broadly, recent reviews emphasize that 3D SERS imaging is advancing through improved nanoprobes, longer-wavelength (NIR) excitation for deeper penetration, and imaging workflows that integrate spatial localization with time-dependent readouts, positioning SERS as a practical tool for spatiotemporal chemical imaging rather than static surface mapping alone [90].

Spatial nonuniformity and temporal fluctuations are tightly connected because the same hotspot structures that produce localized enhancement also govern stability and intermittency. Hotspots that dominate spatial maps are often the same sites where small structural or environmental perturbations cause large time variations. Therefore, a substrate may look spatially uniform in an averaged map but still exhibit strong temporal instability at individual points, and vice versa [73,78].

This coupling motivates reporting SERS performance using both spatial statistics (i.e., map-level distributions) and temporal statistics (i.e., time-series stability) or blinking metrics, especially for quantitative or comparative studies [73,80].

These developments have shifted SERS goals away from maximizing enhancement at a single hotspot toward achieving uniformity, stability, and controlled hotspot occupancy under realistic operating conditions. This shift is evident across dynamic SERS platform development (trapping, responsive materials, wearable hydrogels, intracellular probes), where the design emphasis is often on reproducible spatiotemporal behavior rather than peak enhancement alone.

Accordingly, measurement best practice increasingly combines spatial mapping with time-series acquisition when reproducibility or quantification is required, because either view alone can mask key limitations of the probe or the measurement protocol.

## 2. Fabrication and Microstructure Control of SERS Probes

In this chapter we will overview a few typical methods to fabricate high-quality SERS probes, which include laser ablation in liquid, deposition through self-assembly, laser-direct writing and interference techniques, nanosphere lithography, nanoimprinting, lithographic for large-area substrates and Alumina-anodization oxide methods.

### 2.1. Laser Ablation in Liquids

#### 2.1.1. Principles of Pulsed Laser Ablation in Liquids

Pulsed laser ablation in liquids (PLAL) is a widely used method to produce SERS-active nanoprobes by irradiating solid targets immersed in liquid media [91,92]. This method enables precise control over nanoparticle size, composition, and plasmonic properties, allowing their rational tuning for specific SERS applications. To produce colloidal suspensions of nanoparticles (NPs), one can ablate solid single- or multi-element targets/films immersed in a liquid with a pulsed laser [93,94] or fragment dispersed microparticles, followed by further centrifugation [95,96]. Importantly, PLAL enables the synthesis of SERS nanoprobes under surfactant-free and chemically clean conditions, which is critical for reproducible Raman enhancement and reliable analyte adsorption.

The PLAL process is commonly described as a sequence of several physical stages, encompassing laser–matter interaction, plasma and cavitation phenomena, and subsequent NP formation [97]. The first stage starts with absorbing an incident laser pulse by (i) a solid target and (ii) a surrounding liquid. It leads to rapid material ablation and the formation of a dense plasma plume composed of target species. The second stage is followed by the plasma and cavitation dynamics stage, where the expansion of the plasma generates shockwaves and transfers energy into the surrounding liquid. At the third stage, rapid plasma cooling and heat dissipation induce the formation of a cavitation bubble, whose expansion and subsequent collapse produce secondary shockwaves and strongly affect nanoparticle growth conditions. At the final stage, nanoparticles nucleate, grow, and are released into the liquid environment, forming a stable colloidal suspension.

In this context, nanoparticle nucleation and aggregation during the cavitation stage play a key role in defining interparticle gap distributions, which directly determine hotspot formation. For gaps larger than approximately 1–2 nm, classical electromagnetic theory predicts a strong increase in local field intensity with decreasing gap size. However, at sub-nanometer separations, nonclassical effects such as electron tunneling and charge transfer can reduce field confinement, marking the transition to the quantum plasmonic regime discussed in Section 1.

**Figure 4:**
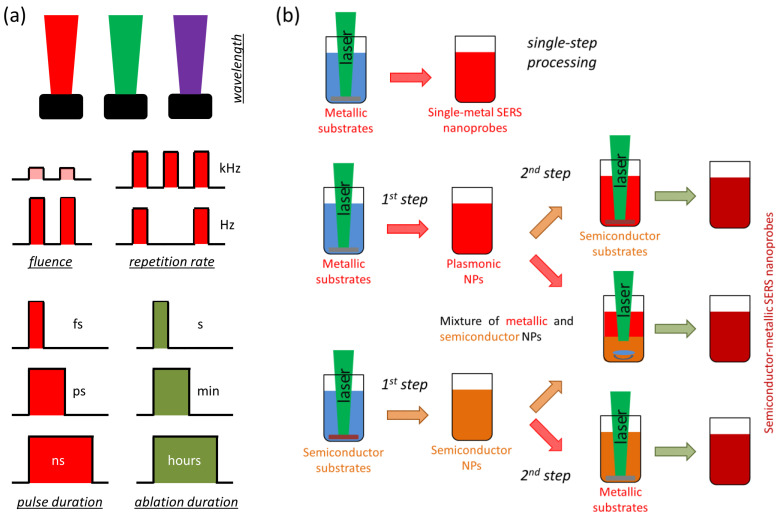
Fundamental principles of pulsed laser ablation in liquids (PLAL) for SERS nanoprobe fabrication: (**a**) key experimental parameters and (**b**) main physical stages.

To synthesize multi-element nanostructures, one can employ the following PLAL approaches (Figure 4):

(1) Ablation of a second target immersed in colloidal suspension prepared by ablation of a first target;
(2) Mixing of several colloidal solutions prepared by ablation of several targets, followed by further laser treatment;
(3) Ablation of a multi-element target (e.g., metallic alloy);
(4) Ablation of a solid target immersed in salt-containing (e.g., AgNO_3_; HAuCl_4_ etc.) solution.

The physicochemical properties and plasmonic performance of the laser-fabricated NPs are governed by a combination of interdependent experimental parameters (Figure 4). They can be broadly classified into laser-related (wavelength, pulse duration, repetition rate, fluence), beam-related (spot size, energy distribution, focusing conditions), and environment-related factors (irradiation time, composition of the surrounding liquid, including salts, polymers, or previously prepared NPs). This provides broad flexibility for developing different kinds of NPs, which can be employed for diverse applications. At the electronic level, these parameters determine the dielectric response function, which governs plasmon resonance conditions, as well as the density of states near the Fermi level. These states near the Fermi level are particularly relevant when charge-transfer contributions to SERS are significant. A wide set of metallic NPs with well-controlled plasmonic properties, which can be directly applied in surface-enhanced Raman scattering (SERS), can be synthesized via PLAL [93,94]. In contrast, pristine semiconductor nanostructures typically lack pronounced localized surface plasmon resonances (LSPR) in the visible range and are therefore not inherently SERS-active. Nevertheless, semiconductors exhibit unique structural, paramagnetic, and optoelectronic properties that are highly attractive for sensing, imaging, photonics, and biomedical applications [98,99,100,101,102,103,104]. As a result, significant efforts have been devoted to enabling or enhancing SERS activity in semiconductor-based nanomaterials.

Laser-induced combination of semiconductors with metallic components provides an effective route to engineer both the optical and electronic properties of SERS nanoprobes, thereby extending their functional range [105,106,107]. The PLAL technique enables the fabrication of multi-metal, multi-semiconductor, and semiconductor–metal nanostructures by incorporating multiple elements within a single nanoparticle [97,105,106,107,108,109,110,111,112]. In such hybrid systems, plasmonic features are either introduced or enhanced in semiconductor nanoparticles, enabling efficient electromagnetic (EM) enhancement, while the semiconductor phase can simultaneously promote charge-transfer (CM) contributions, as discussed in Section 1.1.

A common strategy involves laser ablation of a metallic target (e.g., gold or silver) directly in colloidal suspensions of pre-synthesized semiconductor NPs (e.g., silicon or carbon), prepared by laser-based or chemical methods [110,113]. The efficiency of plasmonic enhancement can be easily adjusted by varying the duration of laser irradiation, which modifies the chemical composition and interfacial structure of the nanocomposites. At the same time, the spectral position of their plasmon resonance is primarily governed by the choice of metallic component [111,113,114].

Compared to conventional wet-chemical synthesis, PLAL offers the advantage of producing ligand-free and chemically clean nanostructures, which improves reproducibility and analyte accessibility. However, this typically comes at the cost of broader size distributions and reduced control over nanoparticle shape and crystallographic facets, which can limit the precise tuning of plasmonic resonances.

In summary, PLAL is a unique tool to design multi-modal SERS nanoprobes at ultraclean chemical conditions.

#### 2.1.2. Multi-Metal SERS Nanoprobes

Multi-metal SERS nanoprobes represent an important extension of laser-based nanofabrication strategies, enabling simultaneous control over plasmonic response, chemical composition, and functional properties. By combining two or more metallic elements within a single nanostructure, it becomes possible to tailor LSPR, improve chemical stability, and introduce additional functionalities, such as magnetic manipulation or catalytic activity.

##### Alloy-Based bi-Metal SERS Nanoprobes (Ag–Au)

Bimetallic Ag–Au SERS nanoprobes with controlled alloy compositions were synthesized via pulsed laser irradiation of Ag–Au alloy targets (Ag–Au 50/50 wt% and Ag–Au 80/20 wt%) immersed in aqueous media containing varying concentrations of KCl [115]. Laser ablation was performed in deionized water (20 mL) with KCl concentrations ranging from 0 to 20 mM using a nanosecond laser (1064 nm, 10 ns, 10 kHz, 7 J/${cm}^{2}$).

The plasmonic response of the resulting bimetallic nanoprobes was governed by both alloy stoichiometry and electrolyte concentration. Nanoparticles produced from the Ag–Au 50/50 wt% target exhibited a systematically redshifted LSPR maximum compared to those fabricated from the Ag–Au 80/20 wt% target. Increasing KCl concentration further modulated the plasmonic peak position, yielding LSPR ranges of 443–459 nm for Ag–Au 50/50 wt% and 415–436 nm for Ag–Au 80/20 wt% nanoprobes [115].

##### Laser-Scribed Bimetallic Ag–Au SERS Nanoplatforms

Laser-scribed SERS nanoplatforms based on bimetallic Ag–Au NPs provide an efficient route for integrating ligand-free plasmonic nanostructures into solid substrates with controlled hotspot architectures. In this approach, Ag–Au NPs with a mean size of ~17 nm were synthesized via PLAL of a bimetallic target in deionized water, yielding stable colloidal solutions with a LSPR peak at ~446 nm [116].

The as-prepared Ag–Au colloids were subsequently incorporated into silicon substrates coated with a thin silver film (~25 nm) using continuous-wave (cw) laser scribing. Immersion of the Si/Ag substrate into the nanocolloid followed by cw laser irradiation at 450 nm enabled NP immobilization and in situ restructuring, resulting in hybrid Ag–Au@Si/Ag SERS platforms [117]. This process promoted the formation of multiscale plasmonic features, including interparticle junctions and nanoscale protrusions, supporting strong electromagnetic field localization and broadband plasmonic response.

##### Tri-Metal Ag–Au–Cu SERS Nanoprobes Fabricated by Shaped-Beam PLAL

Tri-metallic SERS nanoprobes combining Ag, Au, and Cu were fabricated using Bessel-beam-assisted femtosecond PLAL, enabling stable ablation and uniform energy delivery over extended interaction volumes [118]. A multi-metal target prepared by melting and mixing Ag, Au, and Cu (99.9% purity) was ablated in liquid using a femtosecond laser (800 nm, 50 fs, 1 kHz, 27 J/${cm}^{2}$).

The resulting multicrystalline Ag–Au–Cu NPs (mean size 27 ± 3 nm) exhibited a broad LSPR band centered at ~550 nm, positioned between the characteristic plasmon resonances of Au and Cu, along with a pronounced shoulder near 700 nm. These optical features were attributed to compositional heterogeneity, particle morphology, and surface geometry variations [118].

Using three-metal Ag–Au–Cu nanoprobes, the following LODs and EFs were achieved:

- LOD: ~5 µM for RDX and ~0.5 µM for PA;
- EF: ~1.7 × 10^4^ and ~2.4 × 10^5^, respectively [118].

##### Magneto-Plasmonic bi-Metal SERS Nanoprobes (Au–Fe)

Multi-metal SERS nanoprobes combining plasmonic and magnetic functionalities were fabricated via PLAL of sputtered Fe/Au and Fe/Au/Fe multilayer targets [119]. Magnetron-sputtered multilayers with controlled layer thicknesses were ablated in acetone using a picosecond laser (1064 nm, 10 ps, 10 kHz, 1.3 J/${cm}^{2}$), yielding core–shell NPs composed of a gold core coated with iron oxide.

#### 2.1.3. Composite SERS Nanoprobes

##### Composite Si–Au Nanoprobes Synthesized by Two-Step PLAL

Composite silicon–gold (Si–Au) nanoprobes were synthesized using a two-step femtosecond PLAL approach, enabling controlled tuning of NP size and chemical composition. In the first step, colloidal silicon nanoparticles (Si NPs) were produced by laser ablation of a silicon wafer in deionized water (10 mL, 20 mm liquid layer) using a Ti:sapphire laser (800 nm, 110 fs, 1 kHz) at a pulse energy of 150 µJ for 30 min [120]. In the second step, a gold target immersed in the Si NP colloidal suspension was ablated under identical laser conditions, resulting in the formation of composite Si–Au nanoparticles.

The chemical composition and mean size of the nanocomposites were controlled by varying the concentration of Si NPs in the colloidal suspension (0–150 µg/mL) during gold ablation. This strategy enabled the synthesis of Si–Au nanoprobes with mean sizes of ~8.5 and ~14.0 nm and gold atomic fractions of 39% and 63%, corresponding to Si NP concentrations of 45 and 25 µg/mL, respectively [121]. Such compositional tuning provided direct control over the plasmonic response of the hybrid NPs.

SERS nanoprobes containing different amount of Au showed Raman spectra with similar bands with a dominant characteristic band at 737 ${cm}^{-1}$ [120]. In addition, a time-gated SERS detection scheme was implemented for *E. coli* sensing, enabling efficient suppression of autofluorescence and acquisition of SERS spectra at 532 nm excitation.

In both bacterial detection cases, a LOD of 10^5^ CFU/mL was achieved, demonstrating the suitability of composition-tuned Si–Au nanoprobes for biosensing applications [120].

##### Ag-Decorated Si Microspheres Produced by Single-Step PLAL

Silver-decorated silicon microspheres were synthesized via a single-step PLAL process, offering a simple route to hybrid plasmonic–dielectric SERS substrates [122]. In this approach, a silicon wafer was ablated in a mixed liquid medium consisting of isopropanol (9 mL) and AgNO_3_ solution (1 mL), with AgNO_3_ concentrations varied between 50 µM and 10 mM. Laser irradiation was performed using a nanosecond laser (532 nm, 7 ns, 20 Hz, 1.5 mJ per pulse) focused onto the silicon surface with a 20 cm focal length lens.

This process resulted in the formation of large silicon microspheres with an average diameter of approximately 1.1 µm, uniformly decorated with silver nanoclusters. The size of the Ag nanoclusters ranged from 10 to 30 nm and was governed by the AgNO_3_ concentration in the liquid. The resulting Ag–Si hybrid microspheres exhibited strong plasmonic activity and high SERS enhancement, with EFs reaching up to ~10^6^ [122].

SERS performance was evaluated using Rh6G as a probe molecule under excitation wavelengths of 473 and 633 nm. Although plasmonic absorption was weaker at 633 nm, this excitation wavelength yielded a higher SERS signal compared to 473 nm, despite strong dye fluorescence at shorter wavelengths. This behavior was attributed to the strong electromagnetic coupling between irregularly shaped Ag nanoclusters and the high-refractive-index Si microspheres. The interaction induced a redshift of LSPRs, while partial transparency of silicon at 633 nm enabled Fabry–Perot–type optical cavity effects, enhancing multiple excitation and scattering events within individual microspheres [122].

By maintaining laser intensities below 1.5 mW/µm^2^, thermal degradation was avoided, enabling time-resolved SERS measurements that captured the formation and subsequent photodegradation of DMAB over ~60 s. These results highlight the capability of Ag–Si hybrid microspheres for single-particle SERS studies and real-time monitoring of plasmon-driven chemical transformations [122].

##### Ge–Ag and Ge–Au Hybrid Nanoprobes Fabricated Using Bessel-Beam PLAL

Germanium-based hybrid SERS nanoprobes incorporating silver or gold were synthesized using Bessel-beam-assisted PLAL, which offers a deeper focal depth, self-healing beam profile, and invariance of intensity along the propagation axis [123]. In this approach, a germanium wafer was ablated in aqueous solutions containing either AgNO_3_ or HAuCl_4_ salts using a femtosecond laser (800 nm, 50 fs, 1 kHz, 300 µJ per pulse). The use of a Bessel beam enabled stable ablation conditions over extended interaction lengths. Metal salt concentrations were varied between 3 and 10 mM to control NP composition.

This method produced multi-crystalline Ge–Ag and Ge–Au nanohybrids with high metal content, reaching approximately 90.4% for silver and 81.9% for gold [123]. Optical absorption spectra revealed distinct contributions from germanium NPs at 264 nm and pronounced LSPR bands at 415 nm for Ag and 540 nm for Au, confirming the formation of plasmonically active hybrid nanostructures.

##### Laser-Synthesized Tungsten Dichalcogenide Nanoprobes with Plasmon-like SERS Activity

Beyond classical metal–semiconductor hybrids, plasmon-like SERS activity can also be induced in laser-synthesized semiconductor nanomaterials. Tungsten dichalcogenide-based nanoprobes were fabricated via femtosecond PLAL using a high-purity WSe_2_ target immersed in deionized water (2 mL, 10 mm liquid layer) [124]. Laser irradiation was carried out at 1033 nm with a pulse duration of 270 fs, repetition rate of 1 kHz, and pulse energy of 100 µJ. The laser beam was focused onto the target surface using a 100 mm focal length lens, while continuous scanning at 5 mm/s was applied for 10 min to ensure uniform ablation.

The resulting colloidal suspensions consisted of multi-crystalline WSe_2_ NPs exhibiting high chemical stability, as indicated by zeta potentials ranging from −30 to −40 mV. Particle sizes were tuned from approximately 53 nm to 21 nm through post-synthesis centrifugation at 500 and 8000 rpm, respectively. Despite the absence of noble metals, these nanoprobes exhibited effective SERS activity attributed to laser-induced structural defects, high refractive index, and emergent plasmon-like optical behavior.

**Figure 5:**
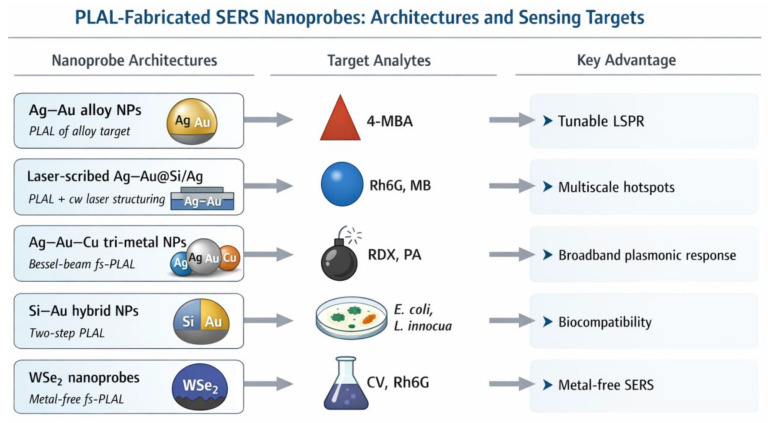
PLAL-fabricated SERS nanoprobes: architectures, representative analytes, and key functional advantages.

**Figure 6:**
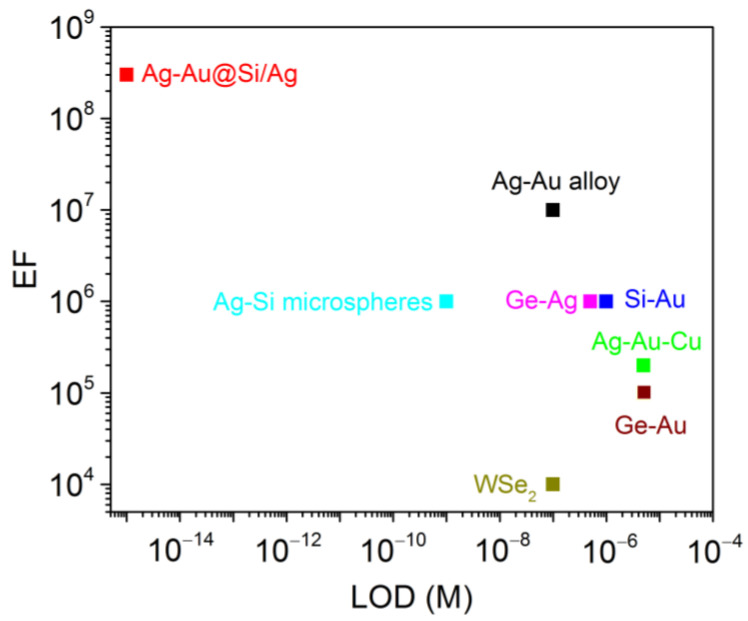
Material-dependent SERS activation mechanisms in PLAL-derived nanoprobes and their sensing targets.

SERS is extensively employed to identify carbon species and molecular structures [125,126]. Serial innovative carbon species, such as polyyne, fullerenes, and hydrogenated poly-carbon, are detected [127,128,129]. Figure 5 presents different types of PLAL-fabricated SERS probes, whereas Figure 6 illustrates the SERS activation mechanisms of PLAL nanoprobes based on different material systems.

#### 2.1.4. High Entropy Alloys (HEA) SERS Probes

##### From Conventional SERS Substrates to High Entropy Alloys

As discussed above, the EM and CT contributions to SERS are governed by the plasmonic response and electronic structure of the substrate, respectively. Silver and gold remain the most widely used materials, but each has practical limitations: silver oxidizes under ambient conditions [5], while gold offers limited electronic tunability at a relatively high cost [4]. Binary and ternary alloys improve on this to some extent, yet thermodynamic miscibility gaps often lead to phase separation and compositional inhomogeneity [130].

**Figure 7:**
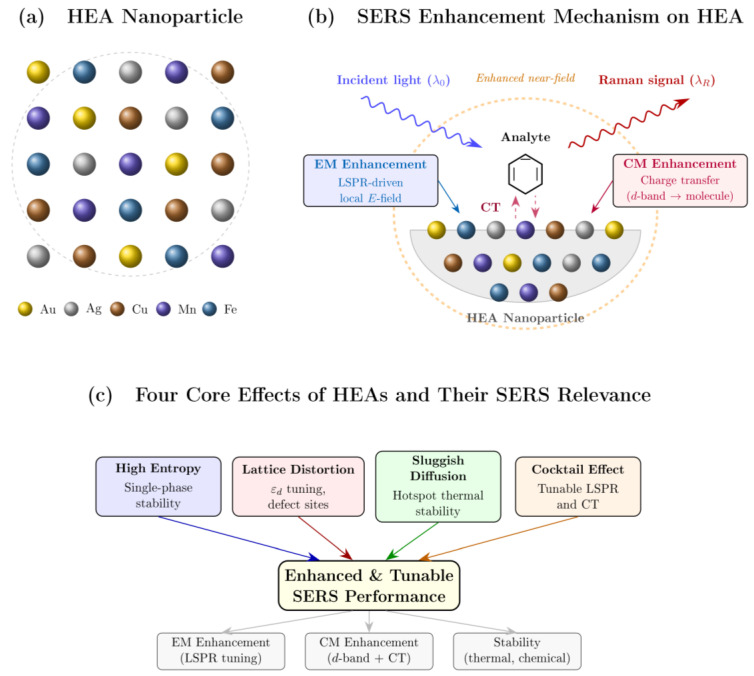
High entropy alloy (HEA) design principles for SERS. (**a**) Schematic of an HEA nanoparticle with five randomly distributed elements. (**b**) SERS enhancement on an HEA substrate. Incident light excites LSPR, producing electromagnetic (EM) enhancement of the local electric field. Concurrently, the tunable d band enables chemical (CM) enhancement through charge transfer (CT) between the surface and the analyte. (**c**) The four core effects of HEAs and their combined contribution to enhanced and tunable SERS performance.

High entropy alloys (HEAs) address both issues simultaneously. Their multi-element composition provides a near-continuous distribution of binding sites and electronic states, so the CT contribution can be adjusted by changing the alloy makeup rather than being fixed by a single metal’s band structure [35,131]. In particular, the resulting “cocktail effect” allows modulation of the *d*-band center to match the electronic requirements of a given analyte, increasing the chemical contribution to the total SERS enhancement [132]. Figure 7a shows the schematic of an HEA nanoparticle with five randomly distributed elements, and Figure 7b shows the SERS enhancement mechanism on HEA Substrate.

This perspective effectively transforms SERS substrate design from discrete material selection to a continuous electronic-structure engineering problem, where the density of states and band alignment can be systematically tuned to optimize molecule–surface interactions.

##### Thermodynamics, Structure, and Electronic Engineering of High Entropy Alloys

The concept of high entropy alloys, introduced independently by Yeh and Cantor in 2004, departed from traditional alloying strategies centered on a single host element [133,134]. HEAs are typically defined as alloys containing at least five principal elements with atomic percentages ranging from 5% to 35% each, and have since found use across mechanical, catalytic, and functional applications [10,135]. The phase stability of an HEA is governed by the Gibbs free energy of mixing (∆*G*_mix_), defined as follows:

(4) $$∆G_{mix}=∆H_{mix}-T∆S_{conf}$$

where ∆*H*_mix_ is the enthalpy of mixing and ∆*S*_conf_ is the configurational entropy [11]. In a random solid solution of *n* elements in equimolar proportions, the configurational entropy is calculated as follows:

(5) $$∆S_{conf}=R\;ln\;n$$

where *R* is the ideal gas constant [136]. For a five-element alloy, ∆*S*_conf_ = *R* ln 5 ≈ 1.61*R*, which is significantly higher than the ∆*S*_conf_ = *R* ln 2 ≈ 0.69*R* found in traditional binary systems [136]. This high entropy term acts as a driving force to stabilize simple crystal structures, such as face-centered cubic, body-centered cubic, or hexagonal close-packed lattices, even when the enthalpic contributions would otherwise favor the formation of brittle intermetallic compounds [133].

The properties of HEAs are commonly attributed to four effects that distinguish them from conventional materials [137]. These effects and their specific relevance to SERS are summarized in Table 1 and in Figure 7c.

Lattice distortion is especially relevant to SERS because atomic size mismatches shift the *d*-band center (*ε_d_*) toward the Fermi level, strengthening molecular adsorption and charge transfer [138,139]. In the Hammer–Nørskov framework, an upward shift of *ε_d_* toward *E_F_* reduces occupied anti-bonding states, strengthening the substrate–molecule bond and promoting charge delocalization [132]. The multi-elemental coordination in HEAs allows *ε_d_* to be tuned over a wide range, potentially circumventing the usual activity–stability trade-off [131]. In AuAgCuMnInBi aerogels, for example, Mn-3*d* orbitals bridge electron-rich elements (Au/Ag/Cu) with In/Bi-*p* orbitals, supporting charge transfer to surface-adsorbed molecules and enabling the detection of crystal violet at very low concentrations [132]. Beyond high enhancement factors, this compositional control provides molecular selectivity by tuning the density of states at *E_F_* to favor the vibrational fingerprints of specific analytes, which is useful for detecting trace pollutants or biomarkers in complex matrices [132,139].

##### Synthesis and Microstructure Control of HEA-SERS Nanoprobes

Fabricating HEA nanoparticles requires non-equilibrium synthesis to maintain atomic-level mixing and suppress phase segregation [10]. A comprehensive review of HEA synthesis strategies is provided by Bridges et al. [135]. The PLAL technique described earlier in this review is well suited to this task: its cooling rates (~1010 K/s) can freeze metastable solid solutions from even immiscible elements. While bi- and tri-metallic PLAL nanoprobes (Ag–Au, Ag–Au–Cu) were discussed previously, extending PLAL to five or more principal elements introduces additional challenges in composition control and phase uniformity [116]. Femtosecond laser-ablated CuCoMn_1.75_NiFe_0.25_ nanoparticles, for example, exhibited plasmonic behavior when the ablation process produced oxide shell architectures, connecting nanostructure control and thermodynamic stabilization to the resulting optical properties [140].

Laser-induced forward transfer (LIFT) offers an alternative route, using femtosecond pulses to deposit HEA microdroplets with minimal thermal damage [141]. CoCrFeNiMo_0.2_ microparticles produced via LIFT exhibit an absorption coefficient *α* ~ 105 cm^−^^1^ at 600 nm and epsilon-near-zero properties suitable for perfect absorbers [141]. Beyond nanoparticle-based synthesis, HEA thin films and bulk structures offer distinct advantages for device integration and large-area reproducibility. HEA thin films have been incorporated into mid-infrared meta surfaces, where their multi-elemental compositions facilitate epsilon-near-zero behavior and precise control over optical absorption coefficients, making them particularly suited for high-performance photonic sensors [142]. For nanoparticle-scale control, wet-chemical approaches such as seed-mediated growth and co-reduction enable HEA atomic layers with tunable facets [132,143]. At the bulk scale, nanoporous quasi-HEA microspheres produced by melt-spinning and selective dealloying, such as Cu–Au–Pt–Pd systems with a surface area of 69.5 m^2^/g, provide an abundant density of hotspots for SERS [144].

Across all fabrication strategies discussed above, a unifying design principle emerges: SERS performance is primarily governed by the ability to control nanoscale field confinement, hotspot density, and molecule–surface electronic coupling, rather than by the specific fabrication method itself.

This principle provides a useful framework for interpreting alternative fabrication approaches, such as self-assembly, where control over gap statistics and hotspot accessibility becomes central.

### 2.2. SERS Fabricated Through Self-Assembly

Self-assembly is a bottom-up fabrication method in which nanoparticles, polymers, and other nanoscale units spontaneously organize into ordered patterns without relying on expensive lithographic tools. For SERS substrates, this approach is attractive because it can create a large number of metal junctions that intensify the local electromagnetic field while remaining compatible with low-cost coating and replication techniques. However, the average performance of a self-assembled SERS substrate depends on more than the presence of a few extreme hotspots. The density and uniformity of the gaps and the ease with which analyte molecules can reach them must also be controlled.

One widely studied strategy is to pack metallic nanoparticles into aggregates or closely spaced monolayers during a controlled drying process. The gaps between particles act as hotspots and can be formed in a single step, but uncontrolled evaporation often produces non-uniform films with broad gap distributions. A more controlled route uses a Langmuir or Langmuir–Blodgett trough to organize particles at a liquid–air interface before transferring the ordered layer onto a solid support. This technique produces two-dimensional nanoparticle lattices whose optical response depends on the particle size and lattice geometry, so the SERS properties can be tuned by choosing appropriate building blocks and assembly conditions [145]. Studies of Langmuir–Blodgett films have shown how the hydrophobicity of the particles, the mechanics of the interface, and the transfer rate affect film uniformity and defect density, which is critical for reproducible SERS measurements [146]. For instance, Tahghighi et al. showed that optimizing the lateral packing and particle size of Langmuir-Blodgett gold nanoparticle monolayers led to a quasi-resonant enhancement condition that maximized the SERS signal of 4-mercaptobenzoic acid [145]. Recent Langmuir–Blodgett SERS films used for rapid screening in complex matrices show that embedding or lightly overcoating the assembled particles can reduce rearrangement during handling while preserving hotspot access [147]. Related work using block copolymer receiving layers demonstrates that constraining particle landing sites can narrow gap statistics and improve map-level uniformity without resorting to full lithography [148]. Langmuir–Blodgett assemblies can also be combined with other materials. For example, gold nanoparticle films transferred onto sheets of molybdenum disulfide are mechanically robust and provide additional interactions that promote adsorption and charge transfer of certain molecules [149].

Template-guided self-assembly uses a pre-patterned scaffold to dictate where nanoparticles land and thus where hotspots form. A notable example combines self-assembled gold nanoparticle clusters with micro- and nano-imprinting into polymer membranes and textiles, producing reusable SERS substrates whose hotspot structure survived repeated detergent-water washing cycles with ultrasound sonication, retaining their SERS activity with no observable degradation of the Au nanoparticle aggregates [150]. In flexible and wearable formats, guided assembly on compliant supports is increasingly used to maintain contact with irregular surfaces while retaining stable nanoscale gaps, which helps translate these architectures to swab and wipe sampling [151].

Porous templating extends this concept into three dimensions. Monodisperse spheres can be assembled into colloidal crystal, infiltrated with a metal precursor, and then removed to leave an inverse opal or macroporous network. The resulting ordered pore lattice serves both as a scaffold for metal deposition and as a photonic crystal that enhances optical field [152]. Alternatively, electrochemical deposition through a sphere template yields highly ordered macroporous gold or platinum films that provide a large surface area with regularly spaced plasmonic features throughout the thickness [153]. Recent inverse-opal SERS arrays that load plasmonic gold nanoparticles onto the internal pore network of silica photonic crystal microspheres have demonstrated simultaneous detection of three mycotoxins (OTA, FB1, and DON) with limits of detection at the pg/mL level, supporting the role of ordered porosity as both an optical structure and a transport pathway [154]. These macroporous architectures are useful when mass transport of analytes into the active region or coupling of optical modes across the substrate is important.

Block copolymers can self-assemble into periodic three-dimensional morphologies such as gyroids, which can then be metallized to produce networks of interconnected metallic ligaments. Gyroid-structured gold substrates provide a high density of hotspots distributed throughout the volume. Wu et al. reported enhancement factors of approximately 10^9^ with relative standard deviations of 5–7% across 30 measurement sites and detection of crystal violet at 10^−10^ M [155]. Reviews of three-dimensional SERS substrates emphasize that bicontinuous networks such as gyroids can reduce spot-to-spot variation by distributing enhancement sites throughout the volume, provided that mapping and reporting include variability metrics rather than isolated best-case spectra [156]. In addition, the three-dimensional network has an optical response that can be tuned by adjusting its dimensions, making it adaptable to different excitation wavelengths.

Nature provides its own templates for self-assembly. The silica shells of diatoms contain intricate periodic pores. When coated with silver nanoparticles they act as hybrid photonic-plasmonic structures. Coupling between localized surface plasmons and guided-mode resonances in the frustule improved SERS sensitivity by a factor of 4–12 compared to nanoparticles on flat glass, with detection of Rhodamine 6 G down to 10^−9^ [157].

Self-assembly can also be combined with semiconducting and photonic components to integrate electromagnetic and chemical enhancement mechanisms. A recent study fabricated a “waffle-like” film by stacking an ordered polymer photonic crystal, a perovskite semiconductor layer, and a thin gold coating. This self-assembled composite achieved detection limits near 10^−10^ M with good signal uniformity and stability, showing how self-assembled templates can host multiple materials to tune optical and electronic properties simultaneously [158].

Programmable self-assembly techniques, such as DNA origami, place metallic nanoparticles at prescribed positions with nanometer-scale precision. Gold nanoparticle dimers assembled on DNA origami with gaps of 3.3 +/− 1 nm produce predictable hotspots with field enhancements of several orders of magnitude, enabling detection of small numbers of dye molecules and DNA oligonucleotides [159]. DNA origami can also align gold nanorods tip-to-tip with gaps of approximately 8 nm, large enough to capture individual proteins while still providing sufficient field enhancement for single-protein SERS with sub-second integration [160]. DNA origami antennas have also been applied to follow single-enzyme activity with single-molecule SERS, illustrating how deterministic assembly can couple controlled gap geometry with controlled target placement [161]. These deterministic assemblies are not yet scalable, but they provide design rules that can inform the engineering of larger-scale substrates.

Quantitative SERS requires that enhancement factors and hotspot distributions be consistent from one measurement to the next. A direct route to such consistency is to define the gap geometry deterministically, such as by inserting nanometer-thick dielectric spacer layers between metal films. Fu et al. fabricated closely packed Ag nanoparticle arrays with 5 nm inter-particle gaps using an ultrathin alumina mask technique, achieving an enhancement factor of approximately 10^9^ and a relative standard deviation of approximately 2% across 10 random spots on a 1 ${cm}^{2}$ [162]. Recent analyses of quantitative SERS outline practical validation steps, including calibration strategies and point-mapping protocols, that are directly enabled by substrates with uniform, accessible gaps [163]. Shell-isolated nanoparticle-enhanced Raman spectroscopy provides a complementary route in which a thin inert shell stabilizes the enhancing metal while keeping near-field coupling, improving comparability when measurements are performed outside tightly controlled laboratory conditions [164]. This result supports an important design principle: instead of chasing rare, extreme hotspots, self-assembled SERS substrates should maximize the number of accessible junctions with controlled spacing and be combined with fluidic and chemical strategies that deliver analytes to those junctions reliably.

### 2.3. Laser Writing and Laser Interference

Ultrafast laser processing, including direct writing and interference lithography, is an attractive pathway to fabricate nanostructures on the target surface, forming various SERS substrates. Theoretically, lasers can process any type of material, no matter how hard, transparent, or fragile. In the fabrication of SERS substrates, the laser beam is generally focused by a high numerical aperture (NA) lens to achieve a spot around 1 μm at the target surface, which realizes the fabrication resolution close to the optical diffraction limit. The high fabrication resolution enables the formation of complicated nanostructures without using masks.

#### 2.3.1. Ultrafast Laser Direct Writing

**Figure 8:**
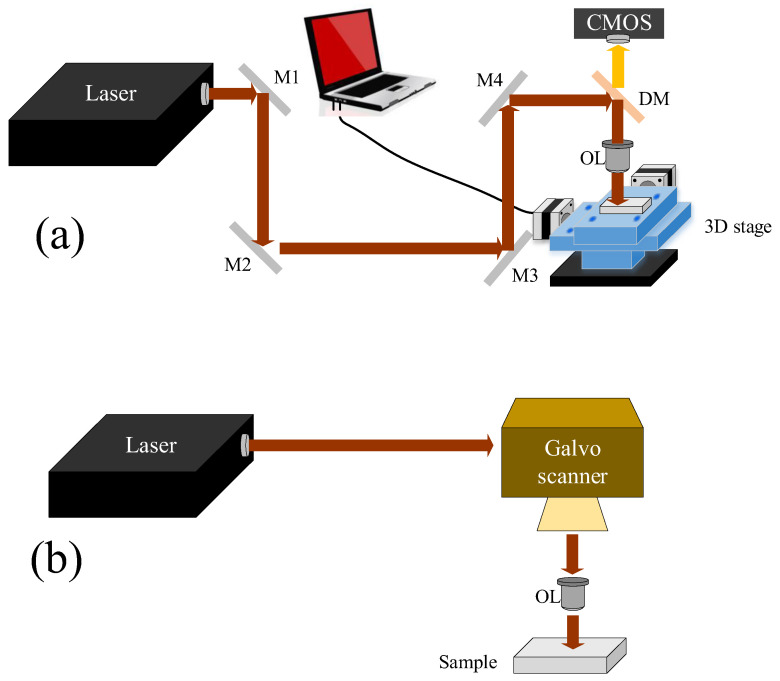
Schematics of laser processing setups for three-dimensional laser direct writing using (**a**) a translation stage and (**b**) a Galvo scanner.

Ultrafast laser direct writing is a typical way to fabricate two-dimensional (2D) and three-dimensional (3D) patterns to form SERS substrates. The patterns are carefully designed to manage the distribution of hotspots on substrates. Generally, dot, groove, pillar, and line patterns can be fabricated according to laser scanning routes on the target surface. To fabricate the patterns, two common experimental setups are preferred: using a motorized translation stage and a galvo scanner, respectively, as shown in Figure 8.

**Figure 9:**
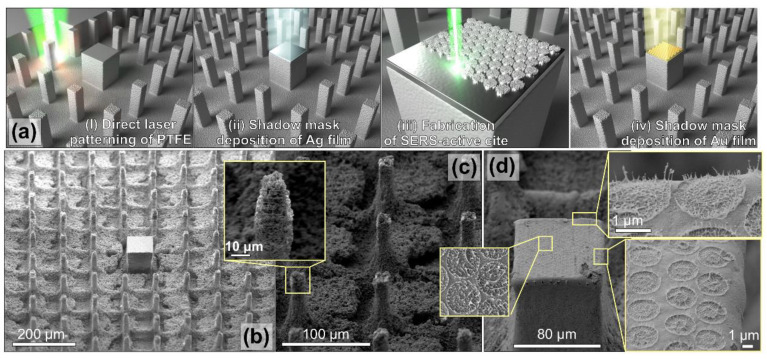
Fabrication of a plasmonic sensor with an analyte-enrichment system. (**a**) Scheme of the sensor fabrication process: (**i**) direct laser writing of analyte-enrichment system on PTFE surface; (**ii**) deposition of 600-nm thick Ag film onto a central pillar through a shadow mask; (**iii**) fabrication of plasmonic sensor element via direct laser texturing of the central pillar; (**iv**) deposition of 25-nm thick Au film through a shadow mask. (**b**) Side-view (view angle of 40°) scanning electron microscopy (SEM) images of the analyte-enrichment system fabricated on the PTFE surface. (**c**,**d**) Close-up SEM images showing details of the surface morphology of the hydrophobic pillars and laser-patterned plasmonic site. Reproduced with permission from MDPI. © 2019 by the authors.

The programmed motorized-3D stage allows the sophisticated patterns to be fabricated on the target surface. Thus, various SERS substrates have been developed to improve SERS performance. Basically, the patterns can be fabricated by laser ablation at the target surface [165]. The substrates, such as glass, were directly scanned by an ultrafast laser to induce a line-structure (groove) with a depth of 5 μm in the laser-irradiated area. Subsequently, silver nanoparticles were deposited on the sidewall and bottom of the groove, forming a homogenous SERS substrate [166,167]. Similarly, the micropillar array was produced on a polytetrafluoroethylene substrate using a femtosecond laser processing system, arranged at a fixed pitch of 95 μm and height of 60 μm. At the top of the pillar array, gold/silver film was deposited by pulsed laser deposition. To smooth the metal film and the morphology, a flat-top beam with sharp shoulders was used, as shown in Figure 9 [168]. The SERS substrate permits organic detection of dye molecules at concentrations down to 10^−12^ M in a reliable manner.

**Figure 10:**
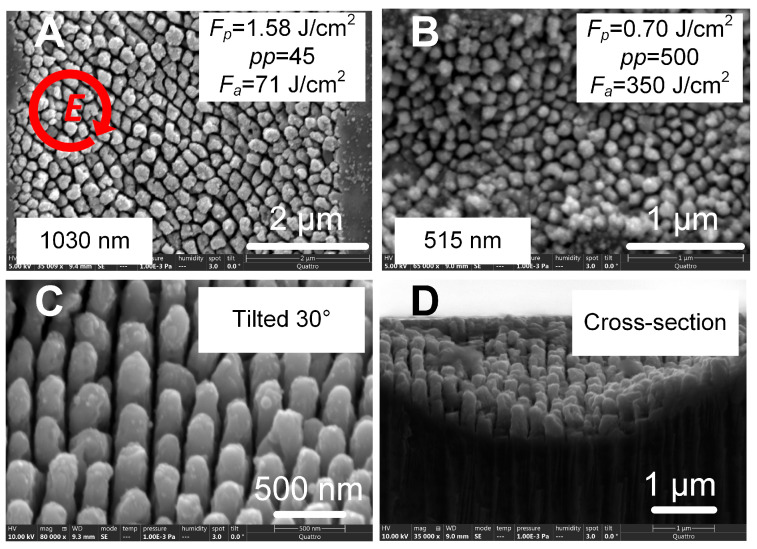
The morphologies of the nanostructure array fabricated by elliptically polarized ultrafast laser scanning with a pulse width of 223 fs. (**A**) *F_s_* = 1.58 J/${cm}^{2}$, *pp* = 45 for 1030 nm. (**B**) *F_s_* = 0.7 J/${cm}^{2}$, *pp* = 500 for 515 nm. (**C**) 30° tilted and (**D**) cross-sectional images of (**A**). *pp*: pulse to pulse overlapping. Reproduced with permission from De Gruyter. © 2023 by the authors.

In recent years, laser reduction of metallic ions and laser-induced periodic surface structures (LIPSS) have become a more attractive approach for SERS substrates fabrication because they provide the possibility to create nanostructures with a feature size beyond optical diffraction. Typically, in laser reduction of metallic ions, silver (Ag^+^) and gold (Au^3+^) ions are prepared in a precursor and then reduced into atoms by absorbing photons in laser irradiation. Subsequently, the metallic atom grows into nanoparticles, and capping agents can be used to control the morphologies of the formed nanoparticles. For example, the SERS substrate was prepared by line-by-line femtosecond laser scanning of silver nitrate solution on a silicon substrate. Thus, the grating-like structure was formed by producing silver nanoparticles. At the concentration of 0.1 M silver nitrate, an enhancement factor of 10^9^ was achieved [169]. Meanwhile, to enhance the efficiency of laser reduction, the gold seeds were used to induce plasmon by laser irradiation [169]. The efficiency could also be directly enhanced by a laser-induced plasma plume on the substrate. For example, via laser irradiation of a silicon substrate, the hydrated electrons derived from the strong ionization of silicon and water contributed to the generation of metallic nanoparticles [170]. LIPSS-formed ultrafast laser processing is another reliable method to create a SERS substrate. For example, periodic nanostructures (generally, groove arrays) were fabricated on a silicon substrate by a femtosecond laser with an 800 nm wavelength and a scan speed of 5 mm/min, forming groove sizes of 165 nm. Subsequently, a 50 nm-thick gold film could be coated on the femtosecond laser-machined substrate to produce SERS substrate [171]. It should be noted that the morphology of fabricated nanostructures is related to the laser processing parameters. The orientation of fabricated grooves is perpendicular or parallel (depending on the material’s properties) to laser polarization, and a dot or pillar array can be created using elliptical polarization [172], as shown in Figure 10, which allows for the fabrication of various SERS substrates to be applied in real applications.

#### 2.3.2. Ultrafast Laser Interference Lithography

**Figure 11:**
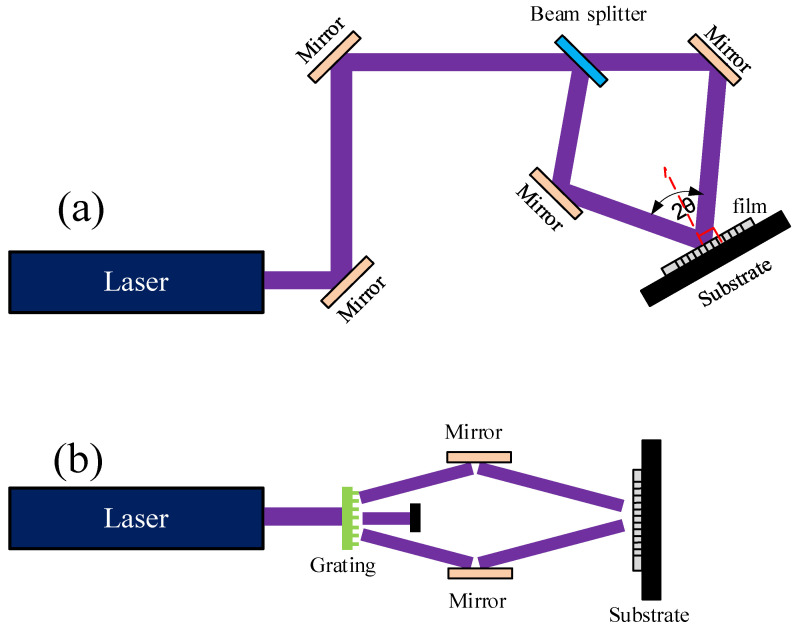
Schematics of laser interferences using (**a**) a beam splitter and (**b**) a grating.

The periodicity of nanostructures is critical for achieving uniform SERS signals, benefiting from a reliable SERS substrate in diverse applications. Laser interface lithography is an efficient method and has been used to fabricate versatile SERS substrates. Generally, a beam splitter or grating is applied for the generation of interfered beams, as shown in Figure 11. The periodicity of the interference pattern is determined by the laser wavelength and the angle of the interfering beams. Thus, a shorter wavelength is preferred to achieve a smaller period, which improves SERS performance.

Mohammadi et al. used a femtosecond laser to write a periodic pattern on photoresist, and silver was deposited on the pattern by a sputter, forming a SERS substrate. A 2D periodic pattern with features on the order of 200 nm was achieved [173]. The laser interference lithography could be involved with other laser processing techniques. Bai et al. applied laser reduction with interference lithography to control the morphology of silver nanostructures. The silver nanospheres and nanoplates could be generated at 365 nm and 616 nm central wavelength light irradiation, respectively, and ~1 nM detection limit of dye molecules was achieved using SERS [174].

### 2.4. Nanosphere Lithography

**Figure 12:**
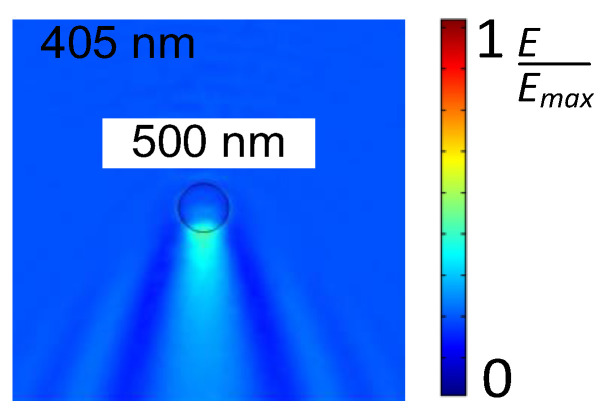
Simulation of a 405 nm laser focused by a 500 nm-diameter silica sphere.

The sensitivity of SERS is directly related to the intensity of the localized electric field, which rapidly decays with increasing distance from the SERS substrate. Thus, fabricating a 1–10 nm gap is essential to acquiring intense SERS signals. However, due to the optical diffraction limit, it is very challenging to fabricate sub-10 nm nanostructures by the aforementioned laser-processing methods. To move beyond the optical diffraction limit, nanosphere lithography is an impressive approach for creating metallic nanostructures with ~10 nm gaps. Generally, the light is focused by a sphere (typically, a silica sphere or polystyrene sphere) with a diameter close to the light wavelength. Then, the electric field is concentrated at the backside of the sphere in a near-field. The radial energy distribution is confined in the near-field, which is smaller than the optical diffraction, as shown in Figure 12.

**Figure 13:**
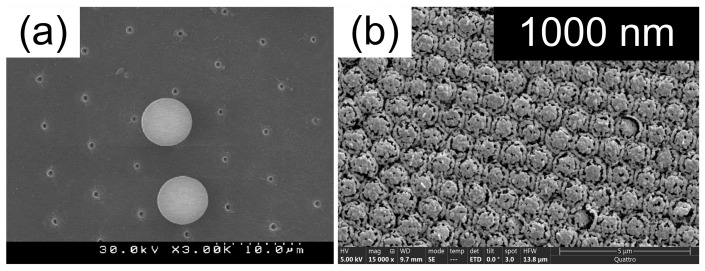
(**a**) SEM image of substrate surface after femtosecond laser (100 fs, 800 nm) irradiation of self-assembly 6.84 μm silica particles. Reproduced with permission from John Wiley and Sons. © 2006 by Elsevier B.V. (**b**) Single-photon absorption induced reduction for fabrication of plasmonic hollow nanocluster arrays with 1000 nm period. Reproduced with permission from John Wiley and Sons. © 2018 by WILEY-VCH Verlag GmbH & Co. KGaA, Weinheim.

Because the light is focused in the near-field at the backside of sphere, the laser processing occurs in the near-field to ablate the target surface. Generally, a monolayer of sphere is used to create periodic structures on the surface, and its period is determined by the diameter of the sphere. Thus, the crater array can be fabricated by laser direct writing, as shown in Figure 13a [175]. Additionally, assisted by spheres, the femtosecond laser near-field reduction can be realized when the multiphoton absorption happens in the near field by carefully adjusting pulse energy. Bai et al. applied the femtosecond laser near-field reduction of silver and gold ions to synthesize plasmonic superstructures for SERS applications, as shown in Figure 13b [176]. In the femtosecond-laser near field, metallic nanoparticles were synthesized at the bottoms of silica spheres and grew along the surfaces of the spheres to form a cage-like structural array. The plasmonic superstructures were used for the detection of perfluorooctanoic acid (PFOA) via SERS, showing a limit of detection of ~11 ppb.

### 2.5. Nanoimprinting

Nanoimprinting in Surface-Enhanced Raman Spectroscopy refers to using nanoimprint lithography (NIL) to fabricate well-defined, nanoscale patterns on a substrate that later act as SERS-active structures. This improves uniformity and reproducibility while keeping fabrication scalable and cost-effective [177,178].

**Figure 14:**
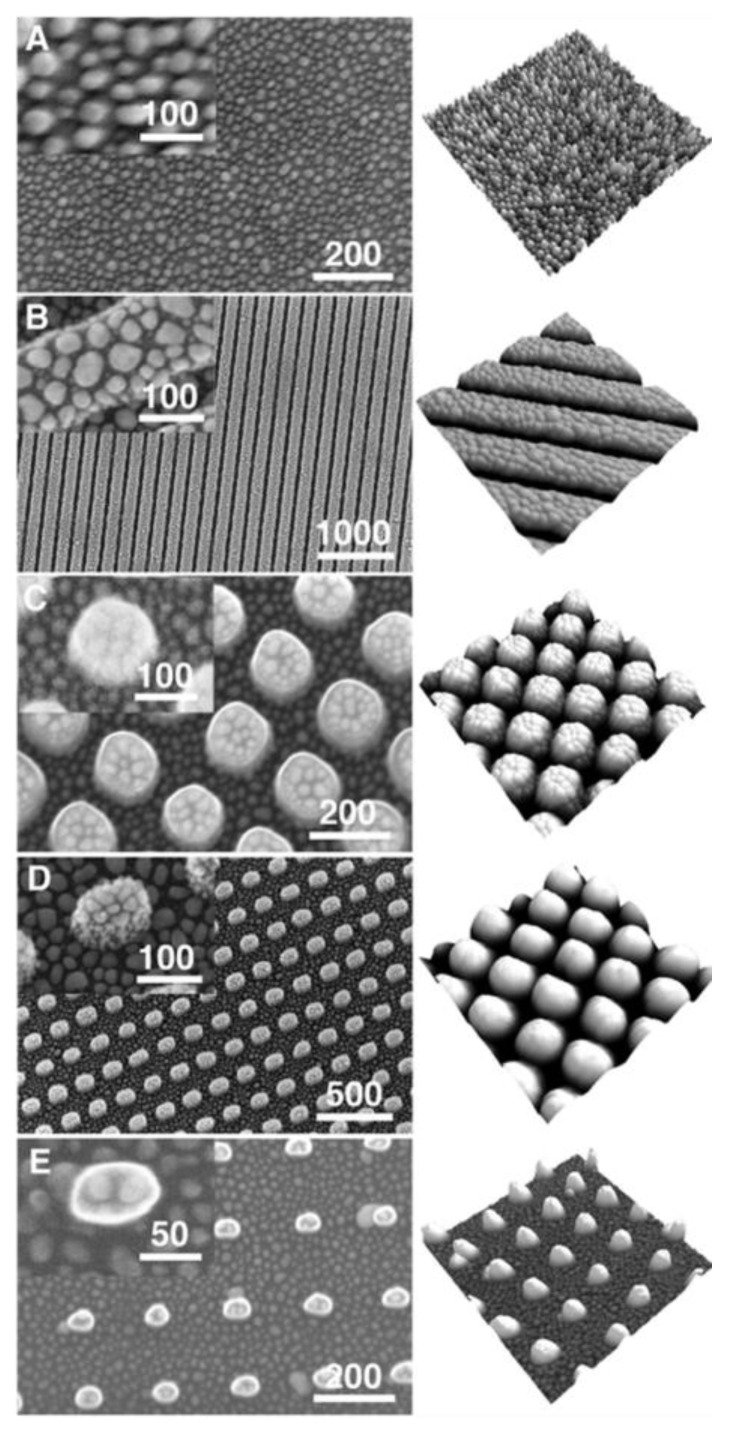
Scanning electron and atomic force micrographs of silver nanoparticles grown by physical vapor deposition on flat (**A**), grated (**B**), and pillared (**C**–**E**) substrates. Reproduced with permission from ref. [179]. Copyright 2007 American Chemical Society.

As shown in Figure 14, Alvarez-Puebla et al. fabricated large-area nanoimprinted SERS substrates followed by Ag deposition, using the imprint geometry to systematically tune the surface plasmon resonance (SPR) while keeping the Ag nanostructure chemistry comparable across samples. Their main contribution was demonstrating that NIL-defined topography provides a deterministic handle to match SPR with the excitation wavelength, improving plasmon–laser coupling and thus SERS output across patterned areas [179].

Chen et al. produced PMMA pillar arrays by NIL (pillar diameter and periodicity varied) and then used a polydopamine-assisted route to grow Ag nanoparticles on the pillars, enabling controlled nanoparticle size and density through growth time. This hybrid “pattern and nanoparticles” strategy improved hotspot consistency and delivered high sensitivity with strong reproducibility, supporting NIL as a route to patterned substrates that reduce spot-to-spot variability in practical SERS measurements [180].

Ferchichi et al. used a two-step NIL replication to transfer Au-coated normal and inverted pyramid arrays from a Si master onto flexible polymer foils, targeting large-area and disposable SERS formats. Both the Si master and polymer replicas provided measurable enhancement, demonstrating that NIL can replicate functional plasmonic topographies onto flexible supports; they also evaluated practical detection using dye/analyte test cases under their fabricated geometries [181].

Colniță et al. fabricated flexible NIL-defined plastic nanotrenches and sputtered nanostructured Ag films (10–100 nm), explicitly linking SERS response to both trench geometry and Ag thickness. Under optimized conditions (reported ~25 nm Ag on nanotrenches), they reported EF up to ~10^7^ and LOD down to 10 pM for crystal violet, highlighting NIL as a scalable route to high-sensitivity flexible SERS with improved uniformity metrics [182].

Badshah et al. developed an Ag-metallized nanoimprinted glass nanohole array designed for high-power laser stability, addressing a common failure mode of polymer-based replicas under high irradiance. They reported that while both glass and polymer replicas reached ~10^7^ EF at 100 mW, the glass substrate sustained much higher-power operation and achieved very large enhancement at 200 mW, whereas the polymer counterpart failed, positioning glass NIL as a route to robust probes for harsher excitation conditions [183].

Garg et al. combined template-assisted self-assembly of Au nanoparticle aggregates (micropatch arrays) with UV micro/nanoimprinting transfer onto membranes and textiles, followed by mild processing to expose hotspots and improve analyte access. A key result was the reusability of the substrate. Detergent-water washing removed the adsorbed analyte while preserving the hotspot structure. No noticeable loss in SERS performance was observed over multiple wash cycles. The substrate also showed enhancement factors on the order of $10^{5}$ and approaching $10^{6}$, supporting its potential for realistic wearable and field-deployable SERS applications [150].

These studies illustrate that NIL provides a practical pathway to microstructure-controlled, reproducible, and scalable SERS substrates, while allowing performance to be engineered through deliberate tuning of geometry, metallization, and mechanical form factor for real sensing environments.

### 2.6. Lithography for Periodic Structures

Lithography-based SERS probes are attractive because they allow the fabrication process to define the active microstructure in a controlled and repeatable way. Instead of relying on random nanoparticle aggregation, these approaches set feature size, periodicity, sidewall geometry, and micro-to-nanogap formation through masks or templates. However, the details of fabrication and the resulting microstructure can strongly affect the magnitude, uniformity, and reproducibility of SERS enhancement.

One widely used benchmark lithographic SERS probe is the Klarite substrate. Klarite-types probes are fabricated through standard silicon microfabrication, where optical lithography defines a periodic pattern, anisotropic KOH etching forms inverted pyramidal cavities, and a thin noble-metal layer, typically Au, is deposited to create the plasmonic surface [184]. The resulting microstructure is a highly ordered pyramid array in which electromagnetic hotspots are expected near sharp corners and edges. Importantly, comparing the standard and next-generation commercial designs showed that using smaller pyramids and placing them more closely together created more field-concentrating sites in the same area. This change improved sensitivity while preserving the good spatial reproducibility of periodic substrates [184].

A follow-up study on the same commercial substrate family connects the same fabrication logic to practical use, including biological testing [185]. The main advantage of this approach is that the fabrication method produces a repeatable geometry, including pyramid pitch, facet angles, and cavity dimensions. As a result, hotspot locations are more predictable, which can reduce spot-to-spot variation compared with stochastic substrates. However, the study also showed an important limitation in biological samples. Large analytes, such as spores or cells, do not always conform well to the cavity structure. Because of this, only a fraction of the analyte may actually reach the high-field regions, which can limit the measured signal even when the substrate itself is structurally uniform. These findings show that an effective microstructure must provide not only sharp features and high hotspot density, but also good hotspot accessibility for the specific target analyte [185]. The sensing performance was evaluated using BPE as a probe molecule and *Bacillus coagulans* spores as a biological sample. The next-generation Klarite 308 achieved the best sensitivity, with BPE detection down to $9.46\times 10^{-13}$ M at 785 nm excitation [185].

Beyond geometry, another study showed that Klarite performance depends not only on how the substrate is fabricated, but also on how the surface changes after fabrication [186]. The substrate uses the same lithography-defined inverted pyramids coated with Au, but the study found that surface aging, oxidation, and contamination can alter the plasmonic response and reduce the enhancement [186]. This means that controlling the microstructure is not enough by itself. The fabricated surface state must also be preserved through proper storage, handling, and measurement conditions. Otherwise, even a well-patterned pyramid array may produce weaker or less reliable SERS signals because of changes in surface chemistry and background fluorescence [186].

Studies using commercial Klarite substrates in complex sample environments still demonstrated the advantages of their engineered microstructured surface. Because the inverted pyramids are fabricated by optical lithography, anisotropic silicon etching, and metal coating, the resulting array has a fixed pitch, uniform cavity dimensions, and reproducible edge and vertex features across the active area [187,188]. This periodic microstructure provides a stable, spatially distributed set of hotspots, so spectra collected from different regions are less dependent on accidentally finding a rare “extreme” hotspot. At the same time, these studies show a practical microstructure limitation. Enhancement depends on how effectively the target molecules reach and adsorb near the high-field regions at the pyramid edges and bottom vertices. This interaction is influenced by how the sample is deposited and distributed across the patterned surface [187,188]. In other words, Klarite’s fabrication produces reproducible hotspot placement, but the effective performance still depends on microstructure accessibility and surface coverage on the pyramid array [187,188].

Overall, lithographic fabrication enables precise control over feature size, periodicity, and nanogap formation, allowing the microstructure to be intentionally engineered rather than randomly formed. As demonstrated across these studies, careful tuning of fabrication parameters directly governs hotspot density, accessibility, and reproducibility, ultimately determining SERS sensitivity and reliability.

### 2.7. Anodic Aluminum Oxide (AAO) with Large Area Self-Assembled Structures

In addition to silicon-based patterned substrates, anodic aluminum oxide (AAO)-based probes represent another important nanomanufacturing platform. AAO probes are fabricated by electrochemical anodization, which differs from photolithography-based methods. Instead of using a photolithographic mask, this approach generates a self-ordered nanoporous template. A foundational study showed that appropriate anodization conditions produce highly ordered hexagonal pore arrays. It also demonstrated that the interpore distance increases approximately linearly with anodization voltage, allowing the pore pitch to be tuned over a wide nanometer-scale range [189]. This matters for SERS because pitch and pore geometry ultimately set how metal coatings or metal-filled structures couple optically, controlling plasmon band position and the density of periodic coupling sites.

**Figure 15:**
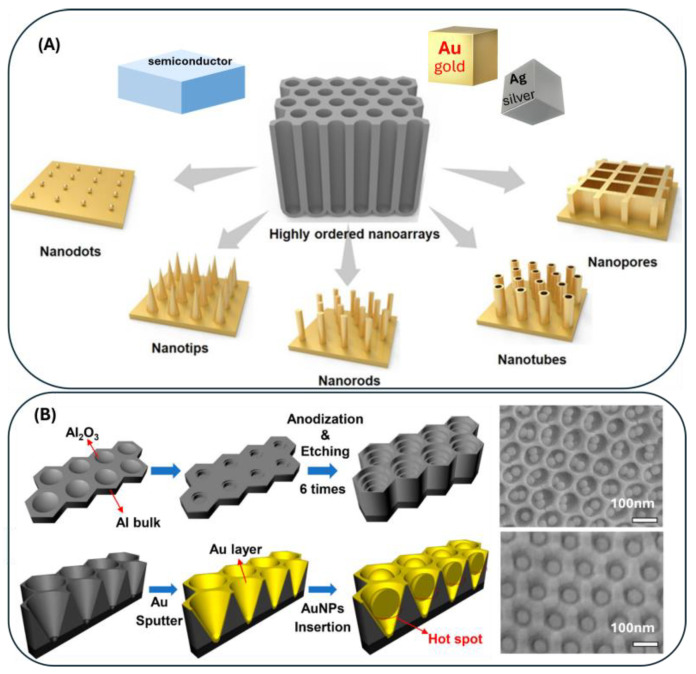
(**A**) Schematic illustration of AAO-template-assisted synthesis of nanostructured arrays with different morphologies. Reproduced with permission from ref. [190]. Copyright 2024 American Chemical Society. (**B**) Fabrication schematic and SEM images of the AuNP-arrayed gold-coated AAO nanostructure. Reproduced with permission from ref. [193]. Copyright 2021 American Chemical Society.

Template microstructure can be further engineered beyond “straight pores” [190]. As shown in Figure 15A [190], a detailed investigation of cyclic processing showed how repeating mild anodization and etching steps can evolve pore profiles from near-cylindrical to parabola-like, trumpet-like, and conical/tapered geometries [190,191]. The fabrication insight is that these cyclic steps allow control over taper angle and opening size, but aggressive etching introduces defects like wall thinning or breaking and taper loss from the top down, which can reduce uniformity. From a SERS design standpoint, sharper or tapered features can strengthen field confinement after metallization, but only if the process window preserves structural integrity and maintains a narrow distribution of feature shapes [191].

A highly SERS-relevant AAO route is to build nanotip and nanorod architectures that intentionally generate sub-10 nm gaps. A previous study reported the fabrication of a conical pore AAO template using repeated anodization and pore widening. This process produced alumina nanotips at the joints of hexagonally arranged pores. Top-view Ag sputtering was then used to form Ag nanorod arrays on the nanotips [192]. The microstructure naturally creates two major hotspot classes: (i) gaps between neighboring Ag nanorods and (ii) gaps associated with rim-deposited Ag nanoparticles. By tuning sputtering duration, the nanorod diameter increases and the inter-rod gap decreases, improving SERS until excessive deposition causes rod overlap that removes accessible nanogaps. This study directly links fabrication control to gap statistics and reports a strong enhancement with good reproducibility under optimized deposition conditions [192].

Another AAO-based design uses conical nanopores to control nanoparticle placement and the metal particle nanogap in a more deterministic way. A conical AAO template was fabricated via a two-step anodization approach, which was modified to create sloped pore sidewalls through repeated short anodization and pore-widening cycles, followed by Au coating and post-treatment to obtain a more continuous Au film [193]. As shown in Figure 15B, Au nanoparticles were then inserted into the conical pores so that the nanoparticle position relative to the gold-coated sidewall is more uniform than in cylindrical pores. The microstructure goal here is to avoid random clustering and instead create controlled “ring-like” coupling regions around each inserted particle, which improves average enhancement and reduces variability compared to uncontrolled aggregates [193].

More recent AAO-derived structures show how the template can be used to create distinct periodic Ag morphologies (e.g., cones vs. claws) by controlling the template depth and metal deposition conditions. One approach deposits thin Ag into V-shaped AAO nanocavities, uses PDMS as a support layer, and then chemically removes AAO to leave a freestanding periodic Ag structure [194]. The resulting microstructures, such as nanocones and claw-like forms, differ in curvature, junction geometry, and local coupling regions. These morphological differences affect hotspot density and field localization, which in turn influence performance. Optimized structures achieve low detection limits for probe molecules and enable biosensing strategies that require reliable enhancement [194].

AAO has also been integrated into multilayer stacks where microstructure control extends into the vertical direction. A composite probe was fabricated by coating AAO with a MoS_2_ interlayer, depositing a continuous Au film, replicating/inverting the AAO structure into a PMMA support by etching away AAO, and then forming Ag nanoparticles by thin-film evaporation conditions that promote islanding [195]. The microstructure is therefore a coupled stack, the inverted AAO-derived topography defines periodic confinement, MoS_2_ modifies interfacial behavior, the Au film provides a conductive/plasmonic base, and AgNPs create dense nanoscale junctions. In this kind of architecture, performance is governed by maintaining uniform layer formation and preventing pore blockage/bridging while maximizing accessible nanogaps across the patterned area, which supports both sensitivity and mapping-level uniformity [195].

To summarize the features of various fabrications of SERS substrates, it is important to point out that each method has its advantages and disadvantages. Laser ablation in liquid is a facile method for nanoparticle synthesis. The precise control of SERS enhancement is difficult. Although local plasmonic resonance can be designed through core-shell structures and by combining different elements, the precision of hotspots positions is unclear. It is natural there are variations in enhancement factors among particles since the hotspots are significantly dependent on the geometry and particle-surface structure. This variation can be mitigated in self-assembled plasmonic structures. However, the precision control of the gap between self-assembled particles is not a trivial task. Laser processing, including direct writing and interferences, can partially control the plasmonic gap. The geometry is also influenced by laser processing, which complicates plasmonic structure control [44,196]. Thermal sintering can also adjust nanogaps between plasmonic nanoparticles [197,198]. Again, thermal sintering is a less controlled process than plasmonic sintering [199].

Nanosphere lithography, nanoimprinting, and photolithography for Klarite substrates represent the latest developments in large-area periodic plasmonic structures. These methods, integrating with atomic design and nanostructure control and enabling multi-level manufacturing across micro-meso-nanoscale, may pave the way for the commercialization of SERS analysis for numerous applications.

## 3. Engineering Optimization of SERS Probes

Although we cover numerous manufacturing methods used to develop highly sensitive SERS probes, the enhancement mechanism is mainly based on EM. In this section, we review metal-oxide and 2D-materials-based SERS probes, which predominantly deploy a CM mechanism or hybrid mechanisms for enhancement. Subsequently, we focus on surface chemistry to improve adsorption and selectivity of analyte molecules. Active ’molecule-escort’ (intelligent molecular targeting) and electrostatic enrichment strategies are further introduced. The role of antifouling in the stability of SERS, and surface engineering for the long-term stability of SERS performance are discussed. To this end, dynamic SERS using microfluidic devices is highlighted as a unique example of hybrid SERS probes with other advanced microsystems.

### 3.1. 2D Materials for Interfacial and Charger Transfer Optimization

Two-dimensional (2D) materials have emerged as important SERS-active and SERS-supporting materials because of their atomically thin geometry, large surface area, tunable electronic structure, and well-defined molecule–surface interfaces. Unlike conventional noble-metal SERS substrates, where Raman enhancement is mainly dominated by electromagnetic field localization at plasmonic hotspots, many 2D materials enhance Raman signals through chemical enhancement, especially via charge transfer between the analyte and the substrate. Their flat surfaces also promote more uniform molecular adsorption, which can improve signal repeatability and reduce hotspot-to-hotspot variability. Therefore, 2D materials are useful both as standalone plasmon-free SERS substrates and as interfacial layers in hybrid plasmonic systems, where they regulate adsorption, charge transfer, fluorescence background, and substrate stability [200,201].

The enhancement mechanism of 2D-material-based SERS can be understood through two main pathways: electromagnetic enhancement (EM) and chemical enhancement (CM). In purely 2D substrates, the EM contribution is usually weak because most 2D materials do not support strong visible localized surface plasmon resonance like Ag or Au. Thus, their Raman enhancement is mainly CM-driven. This occurs when molecule–substrate interactions modify the molecular polarizability and increase the Raman scattering cross-section. Charge transfer is favored when the electronic states of the 2D material, such as the Fermi level, valence band, or conduction band, align well with the HOMO/LUMO levels of the analyte. For 2D transition metal dichalcogenides (TMDs), recent reviews describe this enhancement in terms of static chemical enhancement and resonant chemical enhancement, where charge-transfer resonance, molecular resonance, and exciton resonance can couple through Herzberg–Teller vibronic interactions [202].

Different 2D materials contribute to SERS through different interfacial mechanisms. Graphene can enhance Raman signals through π–π interactions, charge transfer with conjugated molecules, and fluorescence quenching. Its Fermi level can also be tuned by doping or gating to improve energy-level matching with target molecules. h-BN, although insulating, can enhance Raman signals through dipole–dipole interactions originating from polar B–N bonds and can also serve as a chemically stable protective layer in hybrid metal/2D substrates. Other materials, such as g-C_3_N_4_, black phosphorus, MXenes, and TMDs, provide additional tunability through bandgap control, surface terminations, defects, and phase engineering [200,201].

Metallic 2D TMDs are especially promising for plasmon-free SERS because their high density of states near the Fermi level increases electron-transition probability during molecule–substrate charge transfer. For example, ultrathin metallic NbS_2_ was reported as a plasmon-free SERS substrate for methylene blue detection. Compared with graphene, 1T-MoS_2_, and 2H-MoS_2_, NbS_2_ showed stronger Raman enhancement because of its stronger molecule binding and richer electronic states near the Fermi level. Methylene blue was detected down to 10^−14^ M, and the enhancement was mainly attributed to charge transfer rather than conventional visible plasmonic enhancement. Mechanistically, the high density of states in NbS_2_ increases interfacial electron transfer, forming stronger interface dipoles and increasing molecular polarizability [203].

Defect engineering provides another route for optimizing 2D-material SERS probes. Defects, vacancies, edges, and nanopores can act as molecular trapping sites and introduce new electronic states that facilitate charge transfer. Dendritic PdSe_2_ is a representative example, where intrinsic Se vacancies, line defects, dendritic edges, and nanopores serve as plasmon-free SERS hotspots. XPS and atomic-scale imaging confirmed abundant Se vacancies, while DFT calculations showed that defective bilayer PdSe_2_ can develop metal-like behavior. Vacancy-rich PdSe_2_ dendrites achieved an enhancement factor greater than 10^5^ and detected Rhodamine B down to 10^−8^ M. The strongest enhancement occurred at bilayer dendrite edges, followed by monolayer vacancy regions and then the bilayer interior, showing that edge and defect sites are central to the SERS response. In this case, enhancement is mainly CM-based because Se vacancies and edge sites trap RhB molecules and promote charge transfer [204].

Although plasmon-free 2D substrates are promising, their enhancement factors are often lower than optimized noble-metal nanogap systems. Therefore, a practical strategy is to integrate 2D materials with plasmonic metals, combining EM enhancement from metal nanostructures with CM enhancement from the 2D layer. In these hybrid systems, the metal provides strong near-field confinement through localized surface plasmon resonance, while the 2D material regulates molecule adsorption, charge transfer, fluorescence suppression, and interfacial stability [205].

A recent example is femtosecond-laser-induced 1T′/2H-MoTe_2_ nanopatterns decorated with Au nanoclusters. A microsphere-lens-array-assisted femtosecond laser locally converted semiconducting 2H-MoTe_2_ into metallic 1T′-MoTe_2_, and Au nanoclusters were selectively grown on the 1T′ regions. Dimer Au nanoclusters on 1T′/2H-MoTe_2_ produced controlled nanogaps of about 9.2 nm and detected methylene blue down to 10^−13^ M, with an enhancement factor of 4.9 × 10^8^. Here, the enhancement comes from a coupled EM–CM mechanism: Au nanogap hotspots provide strong EM enhancement, while charge transfer between Au, MoTe_2_, and methylene blue further increases the Raman response [205].

Graphene-based hybrid systems also show how 2D materials can tune light–matter coupling. A single Sn nanoantenna on epitaxial graphene enhanced graphene Raman bands by more than two orders of magnitude through localized surface plasmon resonance. When a metallic 2D Sn layer was intercalated beneath quasi-free-standing graphene, the enhancement increased further by forming a nanocavity-like plasmonic interface. The Raman peak shifts indicated that hot-carrier doping, charge transfer, and electron–phonon interactions also contributed to the final response [206].

Overall, 2D materials enhance SERS through tunable CM pathways such as charge transfer, defect states, adsorption, and interface dipoles. When integrated with plasmonic metals, they also support strong EM–CM coupling. Future work should focus on controlling phase purity, defect density, layer thickness, oxidation stability, and molecule-binding sites while integrating 2D materials with ordered plasmonic architectures for sensitive, selective, and reproducible SERS probes.

### 3.2. Metal Oxide Substrates for Chemical Enhancement Dominated SERS Probes

Metal oxides have become an important class of semiconductor-based SERS probes because they offer chemical stability, low cost, biocompatibility, scalable synthesis, and tunable electronic structures. Unlike noble-metal SERS substrates, where enhancement is mainly dominated by localized surface plasmon resonance and electromagnetic hotspots, metal oxide substrates often rely more strongly on the chemical enhancement mechanism, especially charge transfer (CT) between the oxide surface and the adsorbed analyte molecule. This makes them particularly useful for probe optimization, because their SERS response can be adjusted through oxygen vacancies, doping, morphology control, crystallinity, surface functionalization, and heterostructure design. Reported metal-oxide SERS materials include TiO_2_, ZnO, WO_3_, MoO_3_, MoO_2_, Nb_2_O_5_, Ta_2_O_5_, V_2_O_5_, Cu_2_O, CeO_2_, Fe_3_O_4_, and SnO_2_ [71,207].

The main origin of chemical enhancement in metal oxides is the energetic coupling between the semiconductor and the target molecule. When the conduction band, valence band, or defect states of the oxide are properly aligned with the HOMO or LUMO of the analyte, photoinduced charge transfer can occur under laser excitation. This CT process changes the electron density and polarizability of the adsorbed molecule, increasing the Raman scattering cross-section. Possible CT pathways include electron transfer from the molecular HOMO to the oxide conduction band, from the oxide valence band to the molecular LUMO, or from oxide surface/defect states to the molecular LUMO. Because these pathways depend on the band alignment of a specific molecule–substrate pair, metal-oxide SERS can be highly molecule-selective [208].

A central strategy for improving metal-oxide SERS is defect engineering, especially through oxygen vacancies. Oxygen vacancies introduce localized states within the band gap, increase charge-carrier density, and provide intermediate channels for CT [209]. This is important because many wide-bandgap oxides cannot directly absorb visible excitation efficiently. Defect states can also bridge the energy mismatch between the oxide and analyte, allowing visible-light-driven CT. For example, oxygen-deficient TiO_2_, TiO_x_, WO_3−x_, W_18_O_49_, MoO_2−x_, Nb_2_O_5_, and SnO_2_ have shown improved SERS activity due to vacancy-assisted CT. Some oxygen-deficient oxides can also show quasi-metallic or plasmon-like behavior, allowing both chemical and electromagnetic contributions to act together [208,210].

Several metal-oxide examples illustrate this mechanism. Ti-based oxides are widely studied because oxygen vacancies, amorphization, nitrogen doping, and quantum-scale structures can modify the electronic structure and improve CT. ZnO substrates show CT-based enhancement, and their performance can be improved through morphology control, low-temperature annealing, or defect-rich amorphous structures. Mo- and W-based oxides are particularly promising because oxygen vacancies and hydrogen doping can increase carrier density and introduce plasmon-like properties. H_x_MoO_3_, for example, has been reported as a metal-like oxide with strong free-carrier concentration and tunable SERS response. Ta_2_O_5_ nanorods can produce coupled resonance, where local field effects, molecular resonance, and CT resonance act together. CeO_2_ shows facet-dependent SERS behavior because exposed crystal facets influence electron–hole separation and interfacial CT [71,207].

Recent SnO_2_ work provides a clear example of thermally controlled metal-oxide SERS. SnO_2_ nanoparticles synthesized by a sol–gel-assisted route were annealed at 800 and 900 °C. The 800 °C sample showed the best balance between crystallinity, spherical morphology, and oxygen-vacancy density. XPS and NEXAFS confirmed that oxygen vacancies created localized electronic states that promoted CT between SnO_2_ and Nile blue. The optimized SnO_2_ substrate achieved an enhancement factor of 3.95 × 10^3^ and a detection limit of 10^−6^ M, without noble metals or dopants. This shows that even purely non-plasmonic oxides can be made SERS-active through defect-mediated chemical enhancement [211].

Doping engineering provides another important route to enhance CT. Metal or non-metal dopants can introduce sub-bandgap states, reduce recombination, narrow the effective band gap, and improve charge separation. In doped TiO_2_ and ZnO systems, dopants create trap states that allow photoexcited electrons to survive long enough to transfer to adsorbed molecules. This strategy can convert weakly active or inactive oxides into effective SERS substrates. Similarly, morphology control also supports chemical enhancement by increasing surface area and analyte adsorption. Nanorods, nanosheets, aerogels, hollow spheres, porous structures, and nanoflowers with certain surface crystalline orientations, expose more active sites and improve interfacial contact between analyte and oxide [208].

Another emerging approach is heterostructure engineering. Metal oxide@MOF core–shell nanoparticles use a metal oxide core, such as Fe_3_O_4_ or TiO_2_, and a tunable MOF shell to regulate band alignment with the analyte. In RhB detection, Fe_3_O_4_@MOF(Co) and TiO_2_@MOF(Ni) reached enhancement factors of 10^8^ and 10^6^, respectively, with detection limits of 10^−8^ M. Their enhancement was attributed to CT, interband transitions, molecular resonance, and ground-state CT interactions. This demonstrates that metal oxide heterostructures can be rationally designed to improve chemical enhancement by matching the substrate energy levels with the target molecule [212].

Metal oxides provide a versatile platform for chemically enhanced SERS probes. Although their enhancement factors are often lower than those of noble-metal hotspots, their stability, tunability, selectivity, and compatibility with defect, doping, and heterostructure engineering make them highly promising for next-generation SERS probes where reproducible and chemically specific sensing is required.

### 3.3. Surface Chemistry and Functionalization

Surface chemistry plays a central role in SERS because signal generation depends not only on electromagnetic hotspot geometry but also on how analyte molecules are delivered, oriented, and retained within those hotspots. Surfaces are inherently complex systems whose structural, chemical, and electronic properties are difficult to fully characterize and control [213,214]. This complexity is evident in SERS, where signal generation is dependent on plasmonic geometry, particular metal–molecule interactions, and the adsorption of molecules onto the probe surface. To overcome the intrinsic instability of colloidal aggregation, increasing attention has been directed toward engineered nanoparticle assemblies on solid or flexible surfaces. These organized structures provide dense, spatially defined electromagnetic hotspots, which considerably improve signal homogeneity and repeatability [215].

#### 3.3.1. Self-Assembled Monolayers (SAMs)-Driven Hotspot Access

SAMs are essential surface engineering strategies for tailoring the interfacial chemistry of SERS substrates [216], facilitating the controlled and spontaneous arrangement of functional molecules on various types of substrates, such as glass, indium tin oxide (ITO), and silicon [217]. By using salinization [218], thiolation [219], or amine-coupling [220] chemistries, SAMs add certain functional terminal groups (-SH, -NH_2_, -OH, -COOH) on glass, silicon, indium tin oxide (ITO), and metal surfaces. These functional groups regulate nanoparticle attachment, thereby allowing indirect control over nanoparticle density, orientation, and spacing [221]. Well-designed SAMs can introduce molecular spacers that prevent direct quenching while maintaining strong electromagnetic coupling, improving both enhancement reproducibility and spectral consistency, playing a critical role in positioning analyte molecules at optimal distances from the plasmonic surface. Such control is critical for defining hotspot accessibility and ensuring reproducible SERS performance.

**Figure 16:**
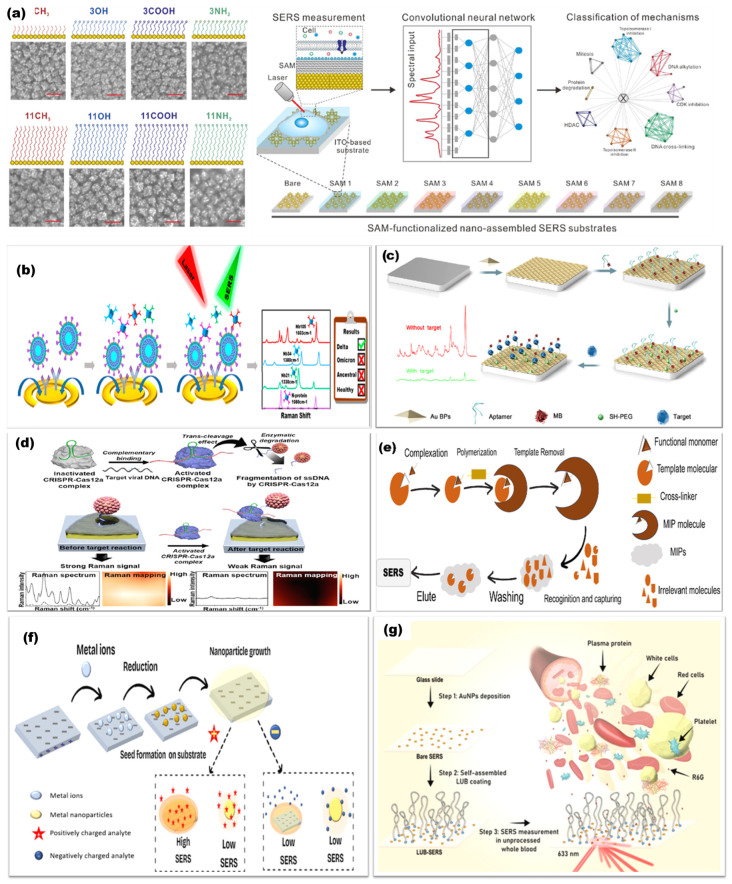
(**a**) A multichannel SERS platform with SAM functionalized surfaces for investigating chemotherapeutic processes, combining various chemical interactions with convolutional neural networks for high precision drug classification. Reproduced with permission from [224]. Copyright 2024, American Chemical Society. (**b**) Antibody-based detection strategy for selective SERS sensing. Reproduced with permission [227]. Copyright 2023, American Chemical Society. (**c**) Aptamer based SERS sensor for specific target recognition. Reproduced with permission from [233]. Copyright 2024, American Chemical Society. (**d**) CRISPR-Cas12a trans cleavage-based sensing mechanism activated by target viral DNA. Reproduced with permission [234]. Copyright 2021, American Chemical Society. (**e**) Molecular imprinting-based SERS sensor for selective analyte recognition. (**f**) Electrostatic enrichment and hotspot formation in plasmonic SERS substrates, where metal nanoparticles capture oppositely charged analytes near electromagnetic hotspots. (**g**) Fabrication of the LUB-SERS substrate and detection of analyte in whole blood, it blocks large molecules while allowing analyte to diffuse and absorb onto nanoparticle to generate SERS signal. Reproduced from [255].

Recent multichannel SERS platforms have exploited heterogeneous SAM chemistries to investigate complex biological systems and drug-treated cells (Figure 16a) [222,223,224], where different surface functionalities promote selective interactions with distinct molecular species. Furthermore, the appropriate selection of head-tail groups enables the functionalization of flexible materials, such as paper, textiles, and adhesive tapes, without affecting mechanical integrity [225]. These SAM-modified, flexible designs enhance nanoparticle adhesion and biomolecular interactions, aiding the development of robust, wearable, and deformable SERS devices [226].

#### 3.3.2. High-Affinity Biorecognition Interfaces

While generic surface functionalization improves analyte accessibility to plasmonic hotspots, high-affinity biorecognition interfaces are essential for achieving molecular selectivity and reliable detection in complex matrices. Rather than increasing analyte concentration in the bulk solution, SAM-based biorecognition interfaces enhance SERS sensitivity by localizing target molecules at the plasmonic interface, thereby increasing the probability that analytes reside within the electromagnetic near-field region where SERS enhancement is strongest.

Antibody-based functionalization represents a classical approach for the specific capture of proteins, pathogens, and disease biomarkers. By selectively binding target molecules, antibody-modified SERS probes enable sensitive detection of pathogens and blood matrix analytes (Figure 16b) [227,228,229]. However, practical limitations, including high cost, limited shelf stability, and susceptibility to denaturation, limit their broader adoption [230].

To address these limitations, functional nucleic acids, including DNAzymes [231,232], aptamers (Figure 16c) [233], and CRISPR systems (Figure 16d) [234], have emerged as alternative recognition strategies, due to their chemical robustness, synthetic flexibility, and tunable binding affinity [235]. Aptamer-functionalized SERS probes selectively immobilize target molecules through sequence-specific interactions. Aptamers are particularly compatible with both solid and colloidal platforms and can be readily integrated into microfluidic and wearable devices. CRISPR-based recognition schemes further expand the toolkit for selective SERS sensing, especially for nucleic-acid targets. In such systems, target-activated cleavage or conformational changes bring Raman reporters into proximity with plasmonic surfaces, converting molecular recognition into a measurable SERS signal.

Molecularly imprinted polymers (MIPs) provide a synthetic, biomimetic alternative to biological receptors. By creating binding cavities complementary in size, shape, and functional groups to the target molecule, MIP-SERS platforms (Figure 16e) selectively retain analytes at the surface and preferentially position them within plasmonic hotspot regions. Their mechanical robustness and chemical stability make MIP-based systems particularly attractive for environmental monitoring and pharmaceutical analysis in complex media [236,237].

#### 3.3.3. Molecular Escort Strategies

While traditional SERS relies primarily on passive diffusion to transport analytes to plasmonic hotspots, recent advancements have introduced a paradigm shift that integrates synergistic physical–chemical enrichment strategies that actively capture, transport, and concentrate target species, a concept central to intelligent molecular targeting. These strategies integrate chemical capture (e.g., aptamers, ligands) with physical concentration mechanisms such as dielectrophoresis (DEP), electrokinetic flow, and microfluidic confinement, enabling directed transport of analytes into plasmonic hotspots. Microfluidic confinement, in particular, increases local analyte concentration by restricting species within nano- to picoliter volumes, while improving mixing, reaction kinetics, and signal reproducibility under controlled flow conditions [238].

Recent systematic analysis by Pei et al. shows that combining chemical affinity, physical confinement, and external force-field manipulation can enhance analyte localization by several orders of magnitude, with overall sensitivity improvements reported in the range of 10^4^–10^15^ depending on system design [239]. These strategies directly address analyte–hotspot decoupling and are particularly effective in complex matrices.

Representative examples include DEP and electrokinetics-assisted SERS [240,241], which concentrates analytes at nanoscale junctions, as well as microfluidic SERS platforms [242], which provide controlled confinement and continuous analyte delivery for improved reproducibility [243]. In addition, electrostatic enrichment systems have demonstrated enhanced local analyte concentration and improved SERS signal intensity in heterogeneous environments [244].

Together, these approaches represent an important extension of intelligent molecular targeting, shifting SERS design from static hotspot engineering toward active control of analyte delivery and enrichment, enabling reliable ultra-trace and single-molecule detection in realistic clinical and environmental samples.

#### 3.3.4. Electrostatic and Interfacial Regulation

Electrostatic and interfacial control are critical to SERS because they affect the adsorption kinetics of analytes on plasmonic surfaces. Charged substrates preferentially attract oppositely charged molecules; as a result, surface charge reversal and the formation of positively charged nanoparticles may dramatically improve sensitivity to dyes, nucleic acids, and ionic contaminants [245,246,247,248]. Electrostatic interactions with charged analytes are often regulated by pH, which alters the negative charge of nanoparticles. Amino acid stabilizers combined with pH adjustment may be used to control the surface charge of Au or Ag colloids to +30 to −50 mV, enabling better capture of positively charged targets [249]. Likewise, negatively charged Ag-deposited Laponite nanoparticles demonstrated selective concentration of cationic probes such as methylene blue and rhodamine B, thereby increasing detection limits (Figure 16f) [244].

Beyond simple charge attraction, amphiphilic SAMs and polymeric coatings provide simultaneous electrostatic interactions, allowing for selective enrichment of targets such as perfluoroalkyl substances (PFAS) at the metal–solution interface [250,251,252]. Rational modification of these interfacial features is thus required to achieve regulated and repeatable SERS performance.

#### 3.3.5. Antifouling and Stability Engineering

In real biological and environmental samples, nonspecific adsorption and surface fouling remain major challenges for reliable SERS detection. Protein adsorption, biofilm formation, and matrix interference can block hotspot access, introduce background signals, and degrade sensitivity over time.

To mitigate these effects, antifouling strategies based on ultralow-fouling zwitterionic SAMs, polymer brushes, and hydrogel coatings have been adopted. These strategies repel nonspecific macromolecules while allowing small analytes to diffuse toward plasmonic hotspots [253], enabling real-time monitoring in undiluted plasma [254]. In complex matrices, such as whole blood and milk, recombinant human Lubricin (LUB) acts as an antifouling coating (Figure 16g) [255]. Janus SERS substrates on flexible textiles offer antifouling, self-cleaning, and directional wettability, allowing for accurate sweat analysis even in high humidity [256]. The shell-isolated nanoparticle-enhanced Raman spectroscopy (SHINERS) [257] represents a complementary strategy, in which plasmonic nanoparticles are encapsulated within ultrathin inert shells. SHINERS preserves strong electromagnetic enhancement while preventing nanoparticle aggregation and has been applied for pesticide detection, environmental monitoring, and biomedical diagnostics, including non-destructive tissue discrimination [258].

#### 3.3.6. Long-Term Performance Stability

High entropy alloy (HEA)-based SERS probes aim to combine high sensitivity with long-term durability. In favorable configurations, HEA probes have reached single-molecule detection levels; an AuFeCoNiCu nanoparticle-based biosensor achieved a 3.4 aM detection limit for miRNA-21 through HEA-catalyzed DNA walker signal amplification [259], while AuAgCuMnInBi aerogels detected uranium at 5 nano Mole (nM) [132]. Enhancement factors for HEA substrates have been reported on the order of 10^6^; Liu et al., for example, calculated an EF of 2.93 × 10^6^ for an optimized Manganese-containing high entropy alloy aerogel (MnHEAA) [132]. Although these values are comparable to those of pristine Au (~10^8^) and below those of Ag (~10^10^), HEAs offer far greater chemical stability [132]. Unlike silver, which degrades through oxidation [5], HEAs containing Cr, Ni, and Co develop a self-passivating oxide layer that protects the metallic core without damping the plasmonic resonance [260]. HEA nanosheets in electrochemical aptasensors retained 96.25% of their initial signal after nine days of storage, a durability attributed to the sluggish diffusion effect that slows hotspot degradation [137,261]. The random atomic arrangement also improves reproducibility: large-area HEA arrays have achieved relative standard deviations below 10%, meeting quantitative analytical requirements [137,262].

SERS also enables in situ monitoring of HEA-catalyzed reactions, including the hydrogen evolution, oxygen evolution, and CO_2_ reduction reactions [131,263]. On CoCuFeMoNi surfaces, SERS has tracked nitrogen reduction and ammonia oxidation intermediates in real time [262]. On AuAgCuPdPt electrodes, in situ SERS combined with DFT revealed that the CO_2_ adsorption configuration governs liquid product selectivity, with the HEA’s multiple transition metal sites achieving a Faradaic efficiency for CO over 96% [264]. More broadly, the structural stability of bulk HEA electrodes under sustained electrochemical cycling provides a combination of high Faradaic efficiency and chemical durability that is difficult to attain with conventional silver or gold substrates [262,264]. Modeling the SERS response of HEAs requires averaging Raman activity over many distinct local atomic environments, each with its own adsorption geometry and polarizability [262,265]. Machine learning approaches, including neural network potentials and DFT-constrained surrogate models, are increasingly being explored to accelerate the screening of HEA compositions for catalytic and sensing applications [139].

### 3.4. Dynamic Analysis with Microfluidics

**Figure 17:**
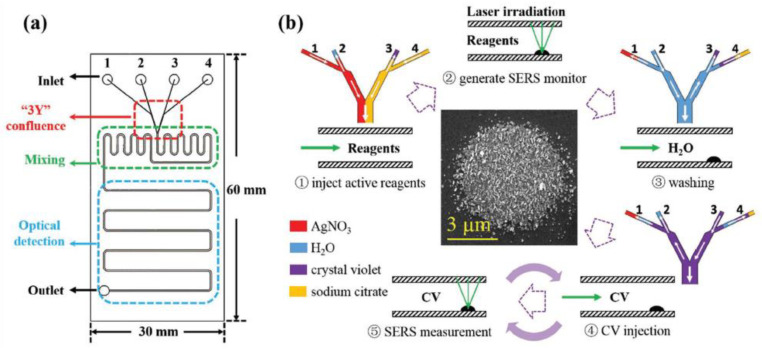
Photo-induced growth of SERS materials and SERS intensity evolution as the sequence of measurement. (**a**) Schematic of a microfluidic chip, with three modules functioning as injection, mixing, and optical detection marked by the red, green, and blue squares, respectively. (**b**) The procedure of photoinduced growth of silver nanoaggregates and in situ SERS measurements. The solutions of AgNO_3_, H_2_O, CV, and sodium citrate in the injection module are represented by different colors. The flow in the microchannel is indicated by the white arrows. Reproduced with permission from John Wiley and Sons. © 2017 by WILEY-VCH Verlag GmbH & Co. KGaA, Weinheim.

**Figure 18:**
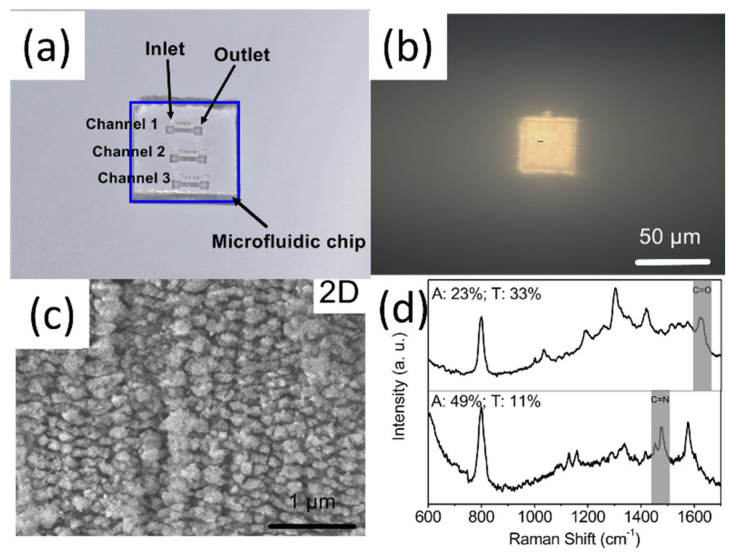
(**a**) Photo of 3D microfluidic chip integrated with SERS substrate fabricated by hybrid femtosecond-laser processing. (**b**) Cu/Ag metal film formed on the laser-ablated region in the microchannel. (**c**) 2D periodic nanostructure produced by LIPSS formation using a femtosecond laser. (**d**) DNA discrimination using a microfluidic SERS chip. Reproduced with permission from John Wiley and Sons. © 2018 by WILEY-VCH Verlag GmbH & Co. KGaA, Weinheim.

Microfluidic SERS chips are an emerging strategy for dynamic analysis in trace detection by SERS. Because the sample is a continuous flow in microchannel, it is usually used as a real-time sensing to monitor a specific analyte. The microfluidic chip enables SERS measurements in a stable environment, which provides reliable SERS signals with superior repeatability and uniformity. Additionally, the microfluidic SERS chip can be coupled with other functional units, such as electrochemical electrodes, to make it more powerful for real applications. Generally, the base material of a microfluidic chip is a polymer (such as polydimethylsiloxane (PDMS)), and molding is used to create microchannels with different shapes and dimensions. Then, the SERS substrate is fabricated in the microchannel or a metallic colloid is used to enhance Raman signals of a specific analyte. For example, Li et al. fabricated a “Y” pattern as an injection module with a mixing module using PDMS. The silver nitrate solution and the analyte solution were injected into the chis through a “Y” injection module, and the silver nanoparticles were created in the mixing module. Subsequently, SERS signals could be detected in the optical detection module, as shown in Figure 17 [266]. Similarly, Liz-Marzán proposed a “T” pattern to mix a silver nanoparticle colloid and analyte solution for remote, real-time monitoring of environmental samples [267]. A syringe pump was used to inject solutions into the channel at a controlled flow rate, and the biomaterials (such as cells) could be identified via the analysis of SERS signals [268,269]. In addition to using metallic nanoparticle colloids, the SERS substrate can be directly fabricated into a microchannel. For example, Sugioka’s group reported a series of studies on the fabrication of microfluidic SERS chips using femtosecond laser processing. The glass microfluidic chip was fabricated by femtosecond-laser scanning of a photosensitive glass (Foturan glass, Schott Glass Corp.). Then, a 30 × 30 μm^2^ region at the bottom surface of the microchannel was ablated by a femtosecond laser, and the Cu–Ag film was formed in the ablated region by electroless metal plating. To create the SERS substrate in the channels, a metal nanodot array is formed on the deposited metal film by double exposure with orthogonal polarization directions of linearly polarized femtosecond laser (orthogonal LIPSS formation) (Figure 18a–c) [270]. Furthermore, the microfluidic SERS chip realized attomole (aM) sensing by focusing the Raman excitation laser at the liquid–air interface in a microchannel, enabling the identification of label-free DNA sequences without polymerase chain reaction (PCR) (Figure 18d) [271]. The microfluidic SERS chip can be coupled with electrochemical analysis function by fabricating electrodes in the microchannel; subsequently, SERS substrate was fabricated on a working electrode [272]. Microfluidic electrochemical SERS chips are generally used for the investigation of redox reactions and are attractive for applications in catalysis and pharmacodynamics.

Recently, a novel technique was developed based on optical trapping/optical tweezers of nanoparticles to form sub-10 nm gap to induce hotspots for ultrahigh sensitivity. Owing to the narrow gap and effective enhancement by hotspot, Raman signal intensity in the capture state was significantly higher than in the non-capture state. In the microchannel, the two nanoparticles can by captured by two trapping lasers; thus, the position and the distance of the two trapped nanoparticles were adjustable from 0 to 600 nm by moving the focal points of the two lasers to form an intense localized electric field in the gap [273]. It has been demonstrated that a single beam can facilitate the aggregation of nanoparticles by irradiating colloids with optical forces. This aggregation of nanoparticles decreases interparticle distances, increasing SERS signals by an order of magnitude. Although this novel technique provides a powerful method even for single-molecule detection (for example, for single-protein detection), it requires complicated devices and highly skilled technicians for measurements and analysis, which remains challenging for real-world application outside of laboratory conditions and in harsh environments.

## 4. Modeling and Machine Learning (ML) for SERS Analysis

Surface-enhanced Raman spectroscopy benefits from a synergistic combination of first-principles modeling and data-driven analysis [274,275]. Density functional theory (DFT) provides quantum-mechanical insight into molecule–metal interactions and chemical enhancement mechanisms, generating theoretical Raman spectra to facilitate peak identification [60,276], while machine learning algorithms extract complex patterns from spectral data and enable automated analysis [275,277]. Rather than competing approaches, these techniques are increasingly integrated: DFT generates synthetic spectra to augment machine learning training datasets [278], and machine learning accelerates or surrogates expensive DFT calculations [278,279]. This section reviews recent advances in both domains and their integration, illustrating how combined theoretical and data-driven strategies are positioning SERS as a quantitative, predictive analytical platform [275,277].

Before reviewing each approach in detail, it is useful to establish the quantitative benchmarks against which modeling and machine learning progress are measured. SERS has historically been evaluated through experimental metrics such as enhancement factor (EF), limit of detection (LOD), and signal reproducibility [280,281]. These following metrics connect computational predictions to the measurable targets.

For modeling to be predictive rather than qualitative, DFT–electromagnetic coupled simulations should aim to reproduce experimental EF values within one order of magnitude [279,280]. Given that typical experimental EF values span 10^6^–10^10^ [282], a computational EF prediction error below 0.5–1.0 log units is a realistic quantitative benchmark [280].

For molecule identification, the relevant metric is the mean absolute error (MAE) between predicted and experimental Raman peak positions: [283]

(6) $$MAE=\frac{1}{N}\sum \left|\omega_{i}^{DFT/ML}-\omega_{i}^{exp}\right|$$

State-of-the-art DFT calculations typically achieve vibrational frequency errors of 5–20 ${cm}^{-1}\;$ after scaling. ML surrogates trained on DFT spectra can reduce effective MAE to below 5–10 ${cm}^{-1}$, approaching experimental spectral resolution [278]. A practical quantitative target for automated SERS identification is MAE < 10 ${cm}^{-1}$.

For ML-driven analyte identification, performance is evaluated via classification accuracy, precision–recall metrics, and confusion matrices [284,285]. In practical sensing applications, a classification accuracy above 90% is generally required for reliable deployment, while high-confidence detection thresholds (e.g., >95% probability) are desirable for environmental and biomedical monitoring. These metrics directly translate computational performance into analytical reliability.

DFT simulations of molecule–surface systems may require several hours to days per configuration. Machine-learning surrogates typically predict spectra in milliseconds once trained. The speedup factor can therefore reach 10^4^–10^6^. For high-throughput substrate or probe screening, a minimum speedup of 10^3^ relative to full DFT is a meaningful quantitative threshold enabling practical compositional exploration.

Machine learning does not directly change the intrinsic electromagnetic enhancement, but it can lower the effective limit of detection by improving signal-to-noise ratios and classification robustness [286]. If denoising and spectral decomposition increase signal discrimination at low intensity levels, the effective LOD can improve by one order of magnitude relative to classical thresholding methods. Thus, computational analysis contributes indirectly but measurably to sensing sensitivity.

From a theoretical perspective, DFT connects directly to the chemical enhancement mechanism by predicting changes in the molecular polarizability tensor alpha upon adsorption. Raman intensity is proportional to the square of the derivative of polarizability with respect to the vibrational normal coordinate Q [287]

(7) $$I\propto {\left|\frac{\partial \alpha }{\partial Q}\right|}^{2}$$

When a molecule adsorbs on a metallic surface, charge transfer and orbital hybridization modify ∂α/∂Q, leading to enhanced Raman activity beyond classical electromagnetic amplification. DFT captures these effects through electronic structure calculations, enabling the prediction of adsorption geometries, charge redistribution, and vibrational mode renormalization.

Machine learning, in contrast, operates at the spectral level: rather than computing electronic structure, ML algorithms learn mappings from spectral patterns to molecular identities or concentrations. In hybrid workflows, DFT provides physically grounded synthetic spectra and mode assignments, while ML adds robustness, scalability, and real-time interpretation capability.

Together, these approaches define a quantitative framework in which modeling predicts the microscopic origin of enhancement and ML translates spectral complexity into actionable analytical decisions.

### 4.1. DFT for Molecule Identification

First-principles approaches have evolved from simple vibrational assignments to comprehensive models of the complex SERS interface, now essential for bridging electromagnetic and chemical enhancement mechanisms [288,289].

#### 4.1.1. First-Principles Modeling: Beyond Classical Electrodynamics

While classical electrodynamics effectively models the $10^{4}$–$10^{7}$ electromagnetic (EM) enhancement at plasmonic hotspots, DFT remains the requisite tool for capturing the “chemical” contribution arising from interfacial electronic coupling [290,291]. This chemical contribution is often interpreted through a unified model involving charge-transfer resonance and the alignment of the metal Fermi level with molecular orbitals [66,292]. Modern SERS modeling has shifted from simple cluster approximations to sophisticated supermolecule treatments that account for charge-transfer (CT) excitations and surface-bonding perturbations.

The recent literature emphasizes a hybrid multi-scale approach: chemical enhancement factors ($EF_{chem}$) derived from polarizability derivatives in DFT are multiplicatively coupled with local field enhancements from finite-element methods (FEM) [10]. For instance, Jensen and colleagues demonstrated that such hybrid frameworks are essential to reconcile the $10^{7}$ total EF observed in benzenethiol-Ag systems, where DFT accounts for the initial $10^{2}$ chemical boost through chemisorption [10]. The current challenge lies in the transition from ground-state DFT to time-dependent (TDDFT) frameworks, which are increasingly necessary to simulate resonance-Raman conditions where laser frequencies align with metal-to-molecule charge-transfer transitions [58].

#### 4.1.2. Validation and Structural Elucidation

The utility of DFT in SERS is best exemplified by its ability to resolve long-standing structural debates. A notable case is the identification of adenine binding on silver; DFT simulations settled conflicting experimental reports by confirming N7-position coordination, identifying the ~733 ${cm}^{-1}$ marker band as a primary indicator for DNA-surface interactions [291].

Similar success is seen in environmental sensing, such as the detection of fipronil and pyridine. By modeling Ag_n_-complexes, researchers have mapped specific ring-breathing mode shifts to charge-transfer excitations, transforming SERS from a qualitative “fingerprinting” tool into a robust method for determining molecular orientation and binding affinity [290,293]. However, discrepancies often arise from experimental variables like substrate morphology and local pH, necessitating iterative refinement of the in-silico metal-surface models to match specific experimental geometries.

### 4.2. ML for Spectrum Analysis

ML methods have become vital in SERS for handling complex, high-dimensional spectral data and extracting meaningful patterns. Unlike traditional univariate analysis (focusing on a single peak or ratio), ML algorithms leverage the full spectral fingerprint, accounting for subtle patterns across many wavenumbers [10,294]. Real-world SERS spectra often contain dozens of overlapping peaks, varying backgrounds, and sample-to-sample variability; ML approaches can navigate this complexity [277,295]. These strategies are particularly critical for biomedical SERS, where the “spectral soup” of biofluids and tissues necessitates advanced chemometrics for clinical translation [296,297,298].

Common preprocessing steps include baseline subtraction (removing fluorescence backgrounds), smoothing and denoising, normalization (scaling intensities), and sometimes spectral alignment (correcting peak shifts) [295]. Cross-validation and independent test sets ensure models generalize to unseen data, addressing the risk of overfitting [295].

#### 4.2.1. Theoretical Foundations of ML/DL for Spectral Data

Before surveying applied architectures, we briefly establish the formal basis of the algorithms invoked throughout this section and motivate why specific algorithmic families match specific characteristics of SERS spectra. SERS spectra possess a distinctive set of statistical properties—high dimensionality (typically 103–104 wavenumber channels), strong collinearity between adjacent channels, sparse informative content concentrated in a small number of vibrational bands, locality and shift-prone nature of these bands, severe baseline interference from fluorescence, and chronic scarcity of labelled data—that select naturally for particular inductive biases. Table 2 summarizes this correspondence; the remainder of this subsection provides the mathematical anchor for each method.

**Linear projection methods (PCA, PLS).** Principal component analysis (PCA) computes the eigendecomposition of the empirical covariance *C* = (1/*N*) *X*^⊤^*X* and projects spectra onto the leading *k* eigenvectors {*v_i_*} that maximise retained variance. Adjacent wavenumber channels in SERS spectra are strongly correlated (peak full-widths typically span 5–20 channels), so most informative variance is concentrated in a low-dimensional linear subspace; uncorrelated shot noise and slowly-varying fluorescence backgrounds populate the discarded components, and PCA acts as an implicit denoiser. Partial least squares (PLS) extends this idea to supervised regression by extracting latent components *t_i_* = *Xw_i_* that maximise cov(*t_i_*, *y*) rather than var(*t_i_*), which makes it the preferred method for concentration estimation on collinear spectra where ordinary least squares overfits. Both methods are linear, however, and degrade when chemically-induced wavenumber shifts cause the same band to populate different feature dimensions—the regime where non-linear manifold methods take over.

**Support vector machines.** A soft-margin SVM solves

min_w,b,ξ_ ½‖w‖^2^ + C Σ_i_ ξ_i_     s.t.     y_i_ (w^⊤^φ(x_i_) + b) ≥ 1 − ξ_i_,   ξ_i_ ≥ 0 (8)

where *φ* is a feature map induced by a kernel *k*(*x*, *x*′) = ⟨*φ*(*x*), *φ*(*x*′)⟩. With a radial basis function kernel *k*(*x*, *x*′) = exp(−*γ*‖*x* − *x*′‖^2^), the decision function depends only on pairwise spectral similarities, allowing the classifier to operate effectively at sample sizes (*N* ≈ 50–200) typical of clinical SERS cohorts where deep networks would overfit catastrophically. The kernel acts as a smooth band-overlap measure; the maximum-margin inductive bias delivers good generalisation in low-data regimes but does not exploit the local 1-D structure of vibrational bands.

**Random forests and gradient-boosted trees.** A random forest (RF) partitions the spectral feature space through an ensemble of decorrelated decision trees, each of which splits on a single wavenumber threshold; bagging and feature subsampling reduce ensemble variance. Gini- or permutation-based feature importances produce a data-driven ranking of discriminative wavenumbers that map onto chemically meaningful vibrational bands and pair naturally with DFT-predicted mode assignments. Because splits depend only on threshold ordering rather than absolute intensity, RFs are intrinsically robust to baseline drift—a robustness deep networks lack without explicit normalisation. Gradient-boosted variants (XGBoost, LightGBM) typically achieve higher accuracy than RF on tabular spectral features at the cost of slightly reduced interpretability.

**1-D convolutional neural networks.** A 1-D convolutional layer computes

y_i_ = σ(Σ_j=−k_^k^ w_j_ x_i+j_ + b) (9)

where *σ* is a non-linear activation. Two properties of this operator align precisely with SERS spectra: (i) locality—vibrational bands are localised features of width ≈ 5–20 ${cm}^{-1}$, so filters with comparable receptive field act as learnable band detectors; (ii) translation equivariance—modest peak shifts induced by chemical environment, substrate-dependent Stark effects, or instrument calibration drift are absorbed by the same filter rather than treated as new features. Hierarchical stacking enables detection of correlated band patterns (e.g., ring-breathing + C–N-stretch co-occurrence) without explicit feature engineering. These two inductive biases are absent from SVM/RF, which is precisely why CNNs dominate when labelled data are abundant (typically *N* > 10^3^).

**Autoencoders and variational autoencoders.** An autoencoder minimises a reconstruction loss as follows:

ℒ(θ, φ) = E_x_ [‖x − g_φ_(f_θ_(x))‖^2^] (10)

through a low-dimensional bottleneck. Trained on noisy ↔ clean pairs (denoising autoencoder), it becomes a learned non-linear baseline-and-noise remover that outperforms parametric baselines, such as asymmetric least squares, when fluorescence is strongly non-stationary. The variational autoencoder (VAE) replaces the bottleneck with a probabilistic latent, optimising

ℒ_VAE_ = E_q_φ(z|x)_ [log p_θ_(x|z)] − KL(q_φ_(z|x)‖p(z)) (11)

which provides a generative latent space and a principled likelihood for anomaly detection—directly relevant to flagging novel analytes or contaminated measurements.

**Generative adversarial networks.** GANs solve the minimax problem

min_G_ max_D_ E_x_ [log D(x)] + E_z_ [log(1 − D(G(z)))] (12)

producing synthetic spectra indistinguishable from the training distribution. In SERS, GANs primarily address the dominant practical bottleneck—labelled clinical and forensic data are scarce—by manufacturing realistic baselines, intensity fluctuations, and hotspot-driven variance that are difficult to model analytically. The standard caveat (mode collapse) is particularly relevant for SERS: a collapsed generator produces non-diverse synthetics that inflate validation accuracy without improving deployment performance. DFT-derived spectra (Section 4.3.1) provide complementary, physically-grounded augmentation that does not suffer from this failure mode.

**Non-linear manifold methods (t-SNE, UMAP).** t-SNE minimises a Kullback–Leibler divergence between Gaussian neighbour probabilities *p_ij_* in spectral space and Student-*t* probabilities *q_ij_* in the embedding:

KL(P‖Q) = Σ_i ≠ j_ p_ij_ log(p_ij_/q_ij_) (13)

preserving local neighbourhoods at the expense of global geometry. UMAP replaces this with a fuzzy-simplicial-set cross-entropy that recovers more global topology while remaining computationally efficient at scale. Both succeed where PCA fails because peak-shift-induced non-linearities cause chemically equivalent spectra to populate distinct regions of the original linear basis but neighbouring regions of the manifold; UMAP is generally preferable for trajectory-like data such as concentration series or time-resolved hotspot dynamics.

**Graph neural networks.** GNNs perform message passing on molecular graphs:

h_v_^(l+1)^ = UPDATE(h_v_^(l)^, AGG{h_u_^(l)^ : u ∈ N(v)}) (14)

which respects permutation invariance over atoms and locality over bonds—the same symmetries that govern vibrational normal modes. This makes GNNs an exceptionally well-matched surrogate for DFT-predicted Raman spectra and enables inverse design of SERS reporter molecules with target spectral signatures.

**Bayesian methods and uncertainty quantification.** Gaussian processes (GPs) and Bayesian neural networks (BNNs) provide calibrated posterior predictive distributions *p*(y*|x*, 𝒟) rather than point estimates. The high-confidence detection thresholds invoked above (≥95% probability for environmental or biomedical deployment) are operationalisable only through methods with calibrated uncertainty; deep ensembles and Monte Carlo dropout offer pragmatic approximations when full Bayesian inference is computationally prohibitive. Conformal prediction provides distribution-free finite-sample guarantees on predictive intervals and is increasingly relevant for safety-critical SERS deployments.

**Transfer learning.** A model pre-trained on source distribution 𝒟_S_ is fine-tuned on target distribution 𝒟_T_ by minimising

ℒ_T_(θ) + λ Ω(θ − θ_S_) (15)

where *Ω* penalises deviation from the source parameters *θ_S_*. This is theoretically justified for SERS because shared low-level features (peak shape, local baseline structure) are largely instrument- and substrate-invariant even when high-level class semantics differ; formally, 𝒟_S_ and 𝒟_T_ share the same conditional *p*(*x*_low_|peak) but differ in *p*(class|*x*_high_).

#### 4.2.2. Supervised Architectures: From Classical Chemometrics to Deep Learning

The transition from classical chemometrics (SVM, RF, PLS) to deep learning reflects the changing data regime of SERS applications: small-*N* supervised problems are well served by kernel and tree methods, whose inductive biases tolerate scarce data, whereas large-*N* tasks benefit from the locality and equivariance priors of CNNs. SVMs remain robust for small-*n* datasets—for example, antibiotic-resistance classification at ≥90% accuracy [295], ref. [281]—while 1-D Convolutional Neural Networks (CNNs) have become the standard for high-throughput diagnostic tasks such as viral detection, where they routinely achieve ≥95% accuracy by exploiting the local, shift-prone structure of vibrational fingerprints described in Section 4.2.1 [294,299,300].

To address the chronic data-scarcity problem in clinical SERS, recent work has pivoted toward Generative Adversarial Networks (GANs) and physics-informed data augmentation. The DeepATSERS framework, for instance, uses GANs to synthesise realistic spectral noise and baseline variations, boosting model generalization for SARS-CoV-2 protein classification from 60% to over 97% [301]. Complementing this, the move toward Explainable AI (XAI)—through class activation mapping (CAM, Grad-CAM) and integrated gradients—ensures that DL decisions correlate with chemically relevant Raman bands rather than instrument artefacts, addressing the long-standing “black-box” criticism of neural networks. The emerging deployment of 1-D vision-transformer architectures, which replace local convolutions with self-attention, extends sensitivity to long-range peak correlations, such as overtone–fundamental relationships, and is beginning to outperform CNNs on multiplexed identification tasks where global spectral context is essential.

In pharmaceutical manufacturing, SERS can monitor reaction intermediates in real time, with ML regression models providing instant concentration readouts. Validation against HPLC showed the ML-SERS approach achieved quantitation within a few percent accuracy while being noninvasive and rapid [302].

#### 4.2.3. Unsupervised Learning: Clustering and Visualization

Unsupervised learning finds patterns in unlabeled data, valuable for exploratory analysis and discovering natural groupings in spectral datasets—applications where label acquisition is prohibitively expensive but where intrinsic structure (e.g., analyte classes, batch effects, novel contaminants) is informative.

**Dimensionality Reduction:** While PCA remains the workhorse for variance-based denoising and orthogonal feature extraction [295], owing to its closed-form eigendecomposition and the direct interpretability of loading vectors as virtual spectra, the field is increasingly adopting non-linear manifold—like t-distributed Stochastic Neighbor Embedding (t-SNE) and Uniform Manifold Approximation and Projection (UMAP)—to resolve the high-dimensional clusters of complex biological analytes where global distance preservation is inadequate [303]. As established in Section 4.2.1, both methods replace Euclidean distance with neighbor-probability metrics that absorb peak shifts and amplitude fluctuations into a local-graph structure rather than treating them as new features.

Applied to SERS spectra of isomeric compounds, t-SNE produces distinct clusters corresponding to each molecule, achieving unsupervised differentiation [304]; t-SNE often separates classes even where PCA projections overlap. UMAP performs comparably on local separation but better preserves global structure, making it preferable for trajectory analyses in single-cell SERS or concentration-dependent series [303].

**Clustering and Anomaly Detection:** K-means and hierarchical clustering partition spectra into groups; spectra within a group are more similar to each other than to other groups [295]. Clustering also facilitates anomaly detection: by modeling normal spectral variability (via PCA, Gaussian mixture models, or VAE), spectra with low likelihood under the normal model flag as anomalous [295]. This is valuable for quality control (identifying bad measurements or contaminations) and discovery (finding novel analytes).

#### 4.2.4. Transfer Learning and Validation

Given that collecting labeled SERS data can be resource-intensive, transfer learning has emerged as a valuable strategy [294,300]. As is formalized in Section 4.2.1, a network pre-trained on a large source corpus (e.g., tens of thousands of Raman spectra from the literature) can be fine-tuned on a smaller target SERS dataset by minimizing *ℒ_T_*(*θ*) + *λ Ω*(*θ* − *θ_S_*). For example, a CNN pre-trained on thousands of Raman spectra from public databases can be fine-tuned with a smaller dataset of clinical SERS samples for a specific diagnostic task [294,300]. The benefit is improved accuracy with limited new data and reduced training time. The justification rests on the substrate- and instrument-invariance of low-level spectral features; mismatched preprocessing pipelines between source and target domains constitute the principal failure mode.

Rigorous validation is critical when adopting advanced models. Standard metrics include accuracy, precision, recall, specificity, $R^{2}$ for regression, and root-mean-square error (RMSE) for quantitation [295]. Internal cross-validation is necessary but insufficient: external validation on independent test sets—ideally collected on different instruments, substrates, and operator cohorts—is required for real-world performance estimation. Nested cross-validation and blind testing on completely new sample sets mitigate overfitting risks [295] and the more insidious leakage risks discussed in Section 4.2.5.

#### 4.2.5. Limitations and Common Pitfalls

The methodological gains surveyed above are accompanied by recurring pitfalls documented across the published clinical and environmental SERS-ML literature. We summarise the most consequential of these to inform critical reading and reproducible practice.

**Substrate-batch confounding.** The dominant failure mode in published SERS-ML studies is spurious correlation with substrate batch rather than analyte identity. When training and test spectra are drawn from the same chips, models learn batch-specific baseline and hotspot signatures that do not transfer; reported accuracies above 99% on internal test sets routinely fall to 60–70% on independent batches. Mitigation requires strict batch separation between training and validation cohorts and reporting of per-batch performance.

**Train–test leakage from co-located spectra.** When multiple spectra are acquired from the same sample location, near-duplicate spectra appear in both training and test partitions under naïve random splits. Sample-level (not spectrum-level) splits are required and, where possible, donor- or experiment-level partitioning.

**Mode collapse in GAN-augmented pipelines.** Synthetic spectra generated by collapsed GANs are non-diverse and produce inflated validation accuracy without improving deployment performance. Generator diversity should be evaluated explicitly (e.g., via Fréchet-distance-equivalent metrics on spectral features) and adversarial augmentation should where possible be supplemented by physics-grounded augmentation from DFT (Section 4.3.1).

**Arbitrary cluster counts in t-SNE/UMAP.** Both methods are sensitive to perplexity (t-SNE) or *n_neighbors_* (UMAP); parameter-induced clusters are commonly mistaken for chemical structure. Cluster validation should be performed across a sweep of these hyperparameters and complemented with stability analysis (e.g., bootstrapped consistency).

**Over-interpretation of feature importances.** RF feature importances are biased toward high-cardinality features and can be unstable across resamplings; permutation importance with confidence intervals should be preferred over default Gini importance, and identified discriminative wavenumbers should be cross-checked against DFT-predicted vibrational assignments before assigning chemical meaning.

**Calibration versus accuracy.** A 95%-accurate classifier that is poorly calibrated may produce confidence scores uncorrelated with the actual likelihood of being correct, undermining the >95%-confidence detection thresholds invoked for environmental and biomedical deployment. Reliability diagrams and expected calibration error (ECE) should be reported alongside accuracy.

### 4.3. Integrated Workflows: DFT-Guided ML and ML-Accelerated DFT

Building on the individual capabilities reviewed in Section 4.1 and Section 4.2, integrated workflows that couple DFT with ML have emerged as a particularly powerful paradigm. DFT-generated spectral data serve as training inputs for ML models, while ML surrogates in turn accelerate costly DFT calculations [300,305].

#### 4.3.1. DFT Spectra as ML Training Data

Building libraries of DFT-computed SERS spectra for many molecules and surface scenarios provides synthetic data for ML algorithms [305]. Xing and colleagues calculated Raman spectra for 166 pesticides using DFT, then used unsupervised learning (PCA/t-SNE) to cluster and identify them by spectral features [304]. The theoretical spectra served as references for ML algorithms to achieve label-free classification of pesticide mixtures without prior training on experimental data [304]. This workflow—DFT as a “digital reference” feeding into AI—exemplifies how DFT’s predictive power can extend SERS reach to analytes lacking experimental standards [304]. This build-up of spectral libraries is foundational for the detection of synthetic chemical pesticides in complex environmental samples [306,307]. Critically, DFT augmentation provides physically grounded variability that complements and avoids the mode-collapse failure mode of GAN-only augmentation discussed in Section 4.2.5.

#### 4.3.2. ML Surrogates for Accelerated DFT

Conversely, ML can accelerate expensive DFT calculations. Hu and colleagues introduced a random forest model trained on thousands of DFT-simulated spectra in various configurations [277,308]. Once trained, the ML model predicted Raman peaks for new configurations nearly instantaneously, with accuracy close to DFT itself [277,308]. Such models enable rapid scanning of how spectra change without full DFT calculations each time.

Graph Neural Networks (GNNs) like Mol2Raman predict Raman spectra directly from molecular structure, having learned the complex structure-to-spectrum relationship from DFT-calculated data [294,300]. As established in Section 4.2.1, the message-passing operation of GNNs respects the permutation and locality symmetries that govern vibrational normal modes, making them an exceptionally well-matched surrogate architecture. These ML surrogates generate spectra orders of magnitude faster than DFT, enabling high-throughput screening of SERS probe candidates and inverse design of reporter molecules with target spectral signatures.

#### 4.3.3. Case Study: Environmental Monitoring Integration

Consider environmental monitoring where SERS substrates detect trace pollutants in water. A deep learning model (e.g., CNN trained on experimental SERS spectra and augmented with DFT-predicted spectra) analyzes each spectrum and outputs the likelihood of different contaminants (pesticides, industrial dyes) along with uncertainty estimates. When the model flags a contaminant with ≥95% confidence—a threshold operationalisable only through the calibrated-uncertainty methods discussed in Section 4.2.1—an alert is generated. Recent advances in co-assembly nanoarrays and AI-driven platforms are further pushing these technologies toward marketable sensors for ultratrace detection [309,310].

### 4.4. Comparison of ML Methods for SERS

Table 3 summarizes representative ML approaches for Raman/SERS analysis, comparing their mathematical core, the inductive bias that makes each suited (or unsuited) to specific SERS data regimes, performance and data requirements, interpretability, and representative use cases. Three trade-offs structure the landscape: (i) classical ML (SVM, RF, PLS) excels with limited data and provides high interpretability but does not exploit the local, shift-prone structure of vibrational bands; (ii) deep learning (CNNs, autoencoders, transformers) achieves the highest accuracy with abundant labelled data through architectural priors that match SERS spectral structure but demands strict validation discipline (Section 4.2.5); (iii) unsupervised methods enable exploratory analysis without labels but require careful hyperparameter validation. Hybrid DFT-augmented and Bayesian approaches occupy a particularly attractive niche, combining physics-grounded priors with calibrated uncertainty estimates that are essential for the >95%-confidence deployment thresholds discussed at the start of this section.

## 5. Emergent Applications

### 5.1. Environmental Applications

#### 5.1.1. PFAS Detection

Per- and polyfluoroalkyl substances (PFASs) have emerged as an important environmental application of SERS because they are highly persistent, present at trace levels in water, and require rapid and portable detection. However, PFASs are difficult SERS targets because they have weak Raman signatures and limited affinity for bare Au or Ag surfaces. As a result, practical PFAS sensing depends strongly on interfacial engineering, with current approaches generally relying on host–guest inclusion, specific interfacial binding, or adsorption-assisted enrichment near plasmonic hot spots.

Among direct strategies, β-cyclodextrin-functionalized Ag nanoparticle probes integrated into microstructure optical fibers provide an important host–guest platform. In this design, β-cyclodextrin captures PFAS in its hydrophobic cavity, Ag nanoparticles generate hotpots, and the optical fiber acts as both a microfluidic channel and a waveguide. This platform enabled direct PFAS detection in water with a detection limit of 40 ng/L for PFOA and also supported differentiation among PFAS species through PCA-assisted spectral analysis [311].

Another direct approach uses an Ag nanoparticle/Au@Ag nanorod sandwich structure, where PFASs are confined within evaporation-induced nanogaps between Ag nanoparticles and a self-assembled Au@Ag nanorod layer. This hotspot-engineered architecture has been used for direct quantitative sensing of PFOA, PFHxA, and PPFBS, with detection down to 0.1 ppm, and is attractive for portable environmental screening because it was demonstrated with a portable Raman device [312].

Recent reviews also show that PFAS SERS probes are expanding beyond these two representative platforms. Host–guest systems now include β-CD/Ag nanocomplexes, Au@MIL-4F core-shell nanocomposites, and other fluorophilic or MOF-based substrates designed to improve PFAS capture near plasmonic surfaces. At the same time, aerosol-jet-printed AgNP/graphene/Kapton substrates, functional porous substrates, and tailored metal–carbon interfaces are being explored to improve adsorption, scalability, and field use [313,314,315].

Indirect PFAS sensing has also shown strong value. In Ag nanocolloid-based dye-assisted methods, PFASs form ion pairs with Raman-active dyes and the SERS signal are measured from the reporter rather than from PFAS directly. A notable example uses methylene blue with Ag nanocolloidal suspensions, enabling the indirect detection of both short-chain PFBA and long-chain PFOA at the 5 ppt level. Earlier related examples include Ag/graphene oxide membranes using dye-based ion pairs and Ag nanoparticle–silica plasmonic superstructures for PFOA detection, showing how indirect sensing can overcome the weak direct adsorption of PFASs on bare metals [14].

**Figure 19:**
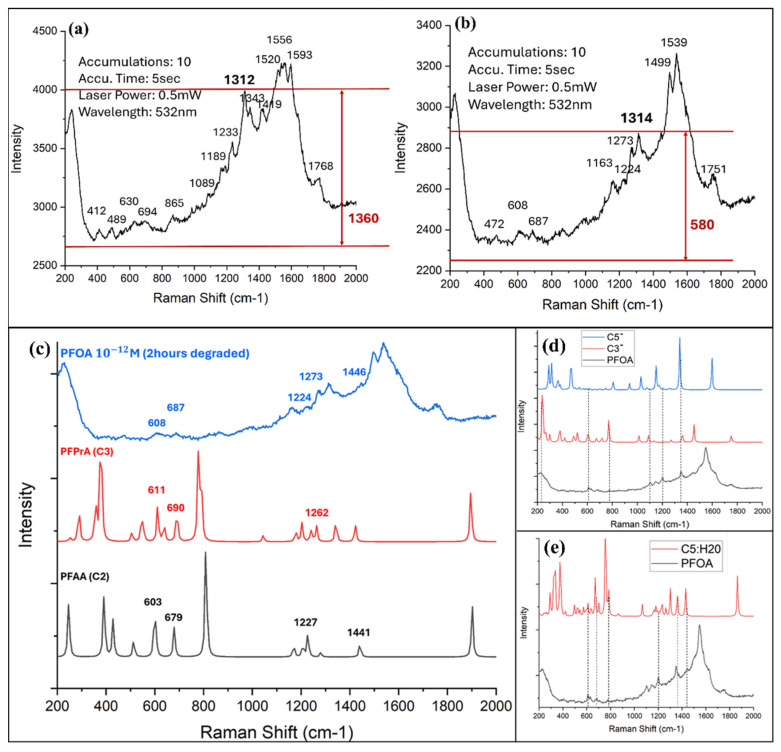
SERS spectra of PFOA ($10^{-12}$ M) before and after photocatalytic degradation. (**a**) Untreated sample showing a prominent characteristic band at 1312 ${cm}^{-1}\;$ with high intensity. (**b**) Spectrum after 2 h degradation, where the 1314 ${cm}^{-1}$ peak decreases, indicating chemical transformation [277]. Comparison of the experimental SERS spectrum of degraded PFOA (10^−12^ M, 2 h) with DFT-calculated Raman spectra of candidate daughter molecules. (**c**) Experimental peaks overlap with predicted bands of shorter-chain products, illustrated by PFAA (C2) and PFPrA (C3). (**d**) Calculated spectra of anionic products compared with PFOA. (**e**) Calculated spectra of water-associated species compared with PFOA. Dashed lines mark representative peak-position matches supporting assignment of degradation intermediates [277].

An additional emerging direction is the use of SERS not only for PFAS detection, but also for degradation monitoring. In a recent manuscript, an fs-laser-fabricated Ag nanoparticle SERS substrate was developed for ultra-trace PFOA detection and real-time monitoring of photocatalytic degradation, as shown in Figure 19. The laser-ablated microchannel confined the Ag nanoparticles to create a high-density hotspot region, and the same probe was used to track spectral changes during degradation, while DFT and machine learning were used to help identify intermediate species [277].

These studies show that PFAS-oriented SERS increasingly relies on application-specific probes, from optical-fiber host–guest systems and sandwich nanogap substrates to indirect dye-assisted colloids and newer hybrid fluorophilic platforms.

#### 5.1.2. Micro- and Nanoplastic Detection

Micro- and nanoplastics have emerged as an important environmental application of SERS because their small size and low abundance make chemical identification difficult, especially in complex samples. Recent studies show that different SERS probe designs are being explored to improve particle capture, signal enhancement, and direct analysis.

One approach uses colloidal Au nanoparticles as the SERS-active probe for microplastic detection in water. In this method, plastic particles are mixed with Au colloids and an aggregating agent before drying on a solid support for Raman measurement. This type of probe produced stable SERS signals for PS microparticles, while PE remained more difficult to detect, showing that colloidal Au systems can be useful for simple aqueous samples but are still polymer dependent [316].

Another study reviews several SERS platforms that have been developed specifically for nanoplastic analysis. These include Ag nanowire arrays and Ag nanowire membranes for filtration-assisted detection, Klarite and Ag-coated SiO_2_ photonic crystal nanowell substrates for single-particle trapping, and AAO-based plasmonic platforms, such as Au nanostar/Ag@AAO and AAO/MoS_2_/Ag particle-in-cavity substrates. In these systems, the main goal is to bring small plastic particles into exposed hot spots or confined cavities where Raman signals can be enhanced more effectively [317].

A Klarite-based study applies SERS to weathered plastics and uses the Au-coated inverted pyramid substrate to identify nanoplastics generated during fragmentation. In this application, the structured surface provides localized hot spots and the grid layout helps relocate particles after SEM imaging, making it possible to chemically identify very small particles after transfer to the Raman microscope. This shows how patterned commercial SERS substrates can be used in environmental weathering studies to confirm the formation of submicron plastics [188].

A different direction uses metal-free AAO membranes as both a filter and Raman substrate for nanoplastic analysis. Instead of adding a metallic SERS layer, the AAO membrane itself is used to capture particles during filtration and then directly analyze them on the same surface. This approach detected standard PS particles down to 200 nm, identified irregular PE, PP, PET, PMMA, PS, and PLA particles, and showed stable performance in solvents, broad pH conditions, and complex matrices such as mineral water and detergent solutions [318]. Another recent approach uses gold nanostar-based SERS tags to label small microplastics on filters. In this method, MBA-functionalized Au nanostars attach to polymers such as PS, LDPE, PET, PTFE, PA6, and PA12, allowing a rapid low-magnification SERS scan to locate tagged particles before Raman analysis confirms the polymer type. This strategy uses SERS mainly as a screening and preselection tool, helping reduce analysis time for small microplastics in environmental samples [319].

These studies show that micro- and nanoplastic analysis by SERS is being advanced through colloidal probes, structured commercial substrates, pore-based plasmonic platforms, and dual-function filtration substrates.

#### 5.1.3. Crucial Mineral Detection

Crucial minerals and metal-related environmental targets have become an important application area of SERS because rapid and sensitive detection is needed in water, food, and environmental samples. In this area, probe design is especially important because some species can be detected directly from their own Raman-active bonds, while others require reporter-assisted or ligand-mediated sensing.

One approach uses Ag-based nanostructured substrates for direct detection of metal oxyanions and oxycations. For example, close-packed films of silver nanocubes, nanocuboctahedra, and nano-octahedra, as well as silver nanoparticle films and self-assembled silver nanowire substrates, have been used to detect arsenate and arsenite through their characteristic As–O vibrations. Similar direct SERS strategies have also been applied to species such as uranyl and neptunyl ions [320].

Another important direction uses functionalized Au or Ag nanoparticle probes for ions that do not generate strong Raman signals on their own. In these systems, DTPA-functionalized gold grating arrays have been used for selective detection of Hg^2+^, Pb^2+^, Cd^2+^, Co^2+^, and Cu^2+^, while GSH/^4^-MPY-modified Ag nanoparticles have been used for As^3+^ detection through analyte-induced aggregation and hotspot formation. Gold nanostars, gold nanohole array chips, and aptamer-modified gold microshells have also been used for highly sensitive detection of Hg^2+^ and Ag^+^, where metal binding changes probe spacing or reporter position and turns on the SERS response [320].

More recent studies also use hybrid and multifunctional nanomaterials to improve selectivity and practical performance. These include Ag–Au core-satellite structures for dual colorimetric/SERS detection of Cu(II), Ag–CoFe_2_O_4_/reduced graphene oxide nanocomposites for Hg^2+^, Fe_3_O_4_-Au@TiO_2_ nanostructures for SERS detection and photoreduction of Cr(VI), and magnetic MOF substrates for rapid and sensitive uranyl detection. Other designs, such as SiO_2_-coated Au–Ag core-shell nanorods, silver telluride nanoparticles, and silicon nanohybrid SERS chips, have been used for targets including Hg^2+^, Pb^2+^, Cr(VI), Mn(II), and Cu^2+^, showing that SERS probe design is moving toward more selective, recyclable, and field-compatible platforms [321].

SERS has also been applied to heavy metal detection in food and agricultural products, where rapid and simple analysis is especially important. In these applications, flexible substrates, paper- or tape-based platforms, microfluidic channels, and chemometric-assisted SERS methods have been used for targets such as Hg^2+^, As^3+^/As^5+^, Cd^2+^, Pb^2+^, and Cr^6+^ in water, rice, tea, and other food-related samples, showing the broader practical value of SERS for metal monitoring beyond model laboratory systems [322].

The broader environmental remediation literature also highlights why such sensing is useful, since treatment of persistent and hazardous contaminants can be costly, energy intensive, and sometimes produce harmful by-products, creating a need for practical monitoring before and after treatment.

### 5.2. Biomedical Applications

The SERS technique has broad applications for biosensing, clinic diagnostics, health care-related devices, and precision medicine [243,296,298]. Integrated with microfluidics, lab-on-a-fiber approaches, and paper-based immunoassays, SERS is extensively deployed for biomolecule identification. Some typical results can be overviewed as follows.

Pathogen detection is key for infectious diseases. Various pathogens, such as bacteria, viruses, parasites, and fungi, have been targets for SERS identification. Label-free SERS detection provides rich molecular fingerprint information from proteins, amino acid residues, and peptidoglycan components of bacterial cell membranes, which can serve as distinctive signatures for bacterial identification. In addition, characteristic SERS spectra may arise from bacterial metabolites, further enhancing detection specificity. Raman mapping is therefore a powerful and widely used approach for bacterial sensing. Integration with microchip platforms—such as microfluidic systems and AC electrokinetic devices—can enable efficient sorting and concentration of bacteria prior to detection. Ultimately, the development of point-of-care SERS systems is highly desirable for applications in food safety and public health monitoring [323].

SERS-based methods enable direct detection of viral capsid proteins, providing a powerful approach for virus identification. For example, dendritic plasmonic nanosensors have demonstrated highly reproducible detection of SARS-CoV-2 RNA under 633 nm excitation (RSD ≈ 3.7%), with ultrahigh sensitivity reaching a limit of detection (LOD) as low as 7.42 × 10^−14^ M [324]. The integration of SERS with lateral flow immunoassays (LFIA) further advances point-of-care diagnostic techniques (POCT). This is exemplified by dual-mode Fe–ZnO@Au–DTNB nanotags for the detection of influenza A (H1N1) [325]. The enhanced sensing performance arises from the strengthened localized surface plasmon resonance (LSPR) of Au nanoparticles, driven by an intensified local electromagnetic field. This enhancement is attributed to efficient charge transfer to Au, resulting from Fe doping in ZnO, which shifts the Fermi level toward the conduction band. Consequently, the system achieves a high enhancement factor (~4 × 10^8^) and a low LOD (0.047 pg/mL), while demonstrating 100% accuracy in clinical H1N1 samples and excellent specificity against other respiratory viruses, significantly outperforming conventional LFIA.

For clinic diagnostics, serum-based SERS platforms integrated with machine learning algorithms are also an efficient method for the early detection of uterine diseases, which is critically important for women’s reproductive health. Here, serum samples mixed with colloidal silver nanoparticles (Ag NPs) demonstrate stable and repeatable SERS spectra using 785 nm excitation [326]. Analysis of 2640 SERS spectra using a one-dimensional convolutional neural network (1D-CNN) successfully and precisely discriminates three distinct uterine diseases from healthy samples. This model achieves a high accuracy rate (93.32%), precision (93.73%), recall (93.99%), and F1 score (93.32%). The Gradient-weighted Class Activation Mapping (Grad-CAM) algorithm improves the reliability and clinical applicability of the results, demonstrating the potential of non-invasive, rapid, and data-driven diagnostics [326].

Detecting neurological disorders accompanied by conformational transitions of neuroproteins is another important application of SERS technology using human saliva. The method is combined with a galvanic molecular entrapment (GME) strategy, entrapping analytes within plasmonic substrates due to the direct formation of hotspots around target molecules. It enhances the detection sensitivity of binary Au–CuO substrates driven by a spontaneous redox reaction between the sacrificial CuO layer and Au ions, allowing the detection and localization of neuroproteins. Such a SERS platform allows the label-free visualization of key proteins involved in the pathogenesis of Alzheimer’s disease—amyloid-β (Aβ42) and tau proteins—in complex biofluids with low LOD ~10^−12^ g/mL [327]. Analysis of human saliva using a logistic regression (LR) model achieves 93.2% sensitivity, 96.7% specificity, and 93.9% accuracy, demonstrating enhanced analyte–hotspot coupling and non-invasive diagnostics of neurological diseases.

Detecting another serious neurodegenerative disease of the central nervous system—Parkinson’s disease—can also be performed using surface-enhanced sensing of serum-derived exosomes [328]. This is provided by a SERS platform based on SP-S1 substrates, whose surface is chemically functionalized with silver nanoparticles (Ag NPs). Using a support vector machine (SVM) algorithm overcomes limitations related to spectral variability and sample heterogeneity that hinder the clinical translation of SERS. Signal heterogeneity during SERS measurements due to the “coffee-ring” effect is mitigated through pre-processing and post-processing, improving detection reproducibility. Thus, it allows the successful discrimination of Parkinson’s disease patients from a control group with an accuracy of 0.85 (95% confidence interval [CI], 0.75–1.00). The study highlights the potential of Ag NP-based SERS nanosensors for early, label-free Parkinson’s disease screening with minimal sample preparation.

Nucleic acid multiplexing detection is the key to developing precision biomedicine. Label-free detection of double-stranded RNA (dsRNA, 19 nucleotides) can be conducted with a SERS platform based on anodized aluminum oxide templates covered with gold layers of different thicknesses ranging from 10 nm to 50 nm. These layers are formed by gold nanodot arrays with interparticle gaps < 10 nm providing efficient electromagnetic field enhancement. It ensures the detection of 10 μM dsRNA concentration on the 50 nm Au nanodot arrays capturing sharp bands at 651 ${cm}^{-1}$ (cytosine), 925 ${cm}^{-1}$ (deoxyribose), 1066 ${cm}^{-1}$ (C−O stretch) and 1238 ${cm}^{-1}$ (uracil) upon 785 nm excitation [329]. Although the sensitivity is not too high (μM range), such SERS nanosensors provide both high reproducibility and surface uniformity accompanied by low-cost production. Magnetic separation can be combined with SERS detection for nucleic acid detection. A magnetic pull-down assisted PCR/SERS assay for the detection of circulating tumor DNA (ctDNA) mutations in the plasma of colorectal cancer patients has been reported [330].

In summary, these studies demonstrate that rational SERS platform design combined with data-driven analysis enables highly sensitive, selective, and clinically translatable SERS biosensing for viral, neurodegenerative, and systemic disease diagnostics. To facilitate the clinical translation of SERS, the validation of its efficacy and safety is required in preclinical large animal models. The detection systems should be optimized to be more feasible, user-friendly, and accessible for clinical practitioners.

### 5.3. National Security

The rise in geopolitical conflicts, combined with the global spread of toxic industrial chemicals, illicit drugs, and improvised explosives, has heightened the need for rapid, field-deployable sensing devices. SERS provides molecular fingerprint specificity and sensitivity required to detect trace explosives, chemical warfare agents (CWAs), and illicit drugs on complex surfaces [331,332,333].

#### 5.3.1. Detection of Explosives

Explosives, including nitroaromatics, organic peroxides, and nitramines, pose substantial hazards owing to their high energy density and tenacity. Engineered plasmonic substrates, such as Ag-ZnO nanoflowers and Au/Ag core-shell nanomotors, can detect substances as low as 10^−14^ M, including trinitrotoluene (TNT) [334] and picric acid [335]. Beyond structural hotspots, electrostatic coordination techniques have proved transformational. For example, alkali-ion-assisted SERS enables the detection of CL-20 and 2,4,6-trinitrochlorobenzene (TNCB) at sub-nanomolar concentrations [336]. In these systems, alkali ions bind to the nitro group’s oxygen atoms to form positively charged complexes that preferentially adsorb on negatively charged silver surfaces. Semiconductor-based systems, including TiO_2_ aerogels with tunable oxygen vacancies, may increase charge transfer by up to 2.42 × 10^−7^ [337].

#### 5.3.2. Chemical Warfare Agents

Chemical warfare agents (CWAs) include a wide range of chemical classes, including nerve agents, vesicants, blood agents, lacrimators, and cytotoxic proteins. Organophosphorus nerve agents (OPNAs) are particularly harmful due to their high toxicity and ease of synthesis. OPNAs are highly hazardous and require rapid SERS-based detection [338,339,340]. Modern interface engineering has focused on confining vapor-phase analytes within electromagnetic hotspots. Recent advancements include gas phase SERS employing silver nanoplates for ppm-level detection of DMMP, mimicking nerve agents such as sarin [341] and fiber-based Au nanoparticle substrates for sub-µgL^−1^ detection of VX and HD [342,343]. Selective functionalization using oxime-based capture molecules, together with the incorporation of sorptive polymer layers and metal-organic frameworks (e.g., ZIF-8), has increased selectivity and sensitivity towards OPNAs near the plasmonic surface [344]. Developing integrated, recyclable plasmonic-sorption systems remains the key goal for achieving practical, field-deployable SERS monitoring of trace-level CWAs.

#### 5.3.3. Detection of Illicit Drugs and Forensic Application

Illicit drugs frequently appear in complex matrices, such as blood, urine, wastewater, and hair, typically at low amounts and in mixes with cutting agents, causing overlapping Raman signals. Nanosilver-based SERS substrates detected fentanyl in blood at 50 pg/mL [345], while liquid–liquid interfacial plasmonic arrays enabled direct urine analysis at 1 ng/mL [309]. Core-shell NH_2_-MIL-101(Fe)@AuNP substrates enhanced fentanyl adsorption and achieved LODs below 3 ng/mL in biological fluids [346]. Wearable and flexible platforms, such as Au trisoctahedra constructed on adhesive tape and incorporated into gloves, enable tramadol detection in serum with LODs of ~69 ng/mL, exhibiting practical forensic relevance [347]. SERS-based wastewater analysis utilizing Ag-decorated diatomaceous films allowed for fentanyl identification at sub-ppb levels, as validated by HPLC-MS [348]. Similarly, Au@Ag-modified fibrous paper substrates enabled community-wide methamphetamine detection using distinctive Raman bands [349]. These improvements show that SERS has evolved from a laboratory device to a valuable tool for community-wide drug surveillance and forensic toxicology. Recent SERS platforms for detecting explosives, CWAs, and illicit drugs are summarized in Table 4.

Despite these advances, the transition of SERS from field-deployable prototypes to trusted national-security and forensic tools requires further progress in standardized substrate fabrication and antifouling surface chemistry to achieve performance reproducibility across manufacturing batches and operating environments. AI-based signal interpretation will play a broader role as part of the sensing ecosystem by enabling cross-instrument calibration, robustness against spectral variability, and reliable interpretation in complex backgrounds. Continued convergence of plasmonic materials, interfacial chemistry, and intelligent data analysis is expected to drive the next generation of deployable SERS sensors for explosives, CWAs, and forensic applications.

### 5.4. Automation Analysis with AI

For practical applications in the real world, automated analysis with AI-SERS is required. Currently, automation analysis with AI-SERS is most attractive in biomedicine, especially for precision medicine to analyze target biomolecules in metabolism and diagnose diseases in their early stages. To achieve automated analysis, AI-SERS is usually applied in microfluidics coupled with a Raman spectrometer to enable sample collection, mixing, reaction, detection, and analysis. In addition, microfluidic can be equipped with other functionalized units such as electrochemical analysis to improve the accuracy of AI prediction.

**Figure 20:**
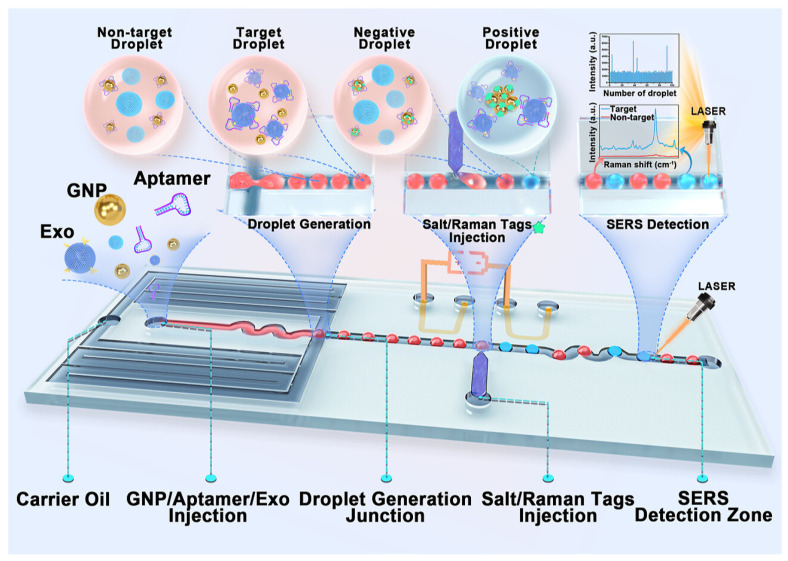
Illustration of the process of a SERS-based droplet microfluidic platform for early cancer diagnosis [355].

Figure 20 shows a typical microfluidic chip coupled with AI-SERS for automated analysis in the application of cancer exosome detection for early cancer diagnosis [355]. The chip involves four primary elements: a droplet generator, an electrode-induced microinjection zone, a curved mixing channel, and a droplet detection zone. The water-based single droplets containing aptamers and exosomes (both extracted from cells), and gold nanoparticles are generated at a water/oil flow-focusing junction. Then, the droplet goes through an injection zone to couple with a Raman reporter in salt solution with the assistance of an applied electric field. Subsequently, the droplet enters a curved mixing channel for SERS signal collection. Owing to on-chip salt-induced gold nanoparticles aggregation, the limit of detection is 4.5 log10 particles/mL, with a high-throughput and a sample-to-result time of 5 min per sample. Chen et al. presented a dual-mode microfluidic platform integrating an electrochemical sensor and AI-SERS to achieve timely and highly reliable quantification of Interleukin-6 in diabetic wound exudates [356]. The electrodes applied in the microfluidic promote the immobilization of analyte molecules on SERS substrate, and both electrochemical and SERS signals are processed by a neural network with a self-attention mechanism. The platform achieves a quantitative accuracy of up to 99.8% across a range of 0.05−1000 pg/mL. Additionally, the AI-SERS platform can be attached to a wearable substrate to monitor human health. For example, Chen et al. demonstrated a wearable AI-SERS platform for detecting uric acid in sweat [357]. The SERS substrate (silver nanowires) is prepared on polydimethylsiloxane (PDMS) and attached to human skin using double-sided medical tape. The sensitive detection of uric acid in sweat is as low as 100 nM and the prediction accuracy of gout is 97% using the ANN algorithm.

## 6. Challenges and Future Perspectives

### 6.1. Detection Limitation and Hybrid Devices

Despite remarkable progress over the past decades of SERS enhancement mechanisms, several critical challenges remain in advancing surface-enhanced Raman scattering (SERS) toward its full analytical potential. First, the spatial and temporal resolutions of current SERS systems are still limited by existing instrumentation. The temporal resolution of conventional SERS measurements is typically at the millisecond level, primarily constrained by the readout speed and sensitivity of charge-coupled device (CCD) detectors commonly used in Raman spectrometers [32]. This limitation restricts the ability to monitor ultrafast molecular dynamics, charge-transfer processes, and transient catalytic intermediates occurring on picosecond or femtosecond time scales. Integrating SERS with ultrafast spectroscopic techniques, such as femtosecond pump–probe Raman spectroscopy and time-resolved plasmonic spectroscopy, may enable the investigation of ultrafast plasmon–molecule interactions and non-equilibrium processes. Meanwhile, due to the optical diffraction limit of conventional far-field optics, the spatial resolution of SERS imaging is generally restricted to several hundred nanometers. Emerging approaches combining SERS with super-resolution microscopy techniques—such as tip-enhanced Raman spectroscopy (TERS), near-field scanning optical microscopy (NSOM), and scanning transmission electron microscopy (STEM)-assisted vibrational spectroscopy—offer promising routes to achieve nanometer or even sub-nanometer spatial resolution. Second, the integration of SERS with complementary in situ and operando characterization techniques represents an important direction for capturing multidimensional information during chemical or biological processes. For example, coupling SERS with electrochemical spectroscopy in spectroelectrochemical cells enables the simultaneous monitoring of interfacial redox reactions, catalytic kinetics, and molecular adsorption dynamics on plasmonic electrodes [358,359]. Similarly, combining SERS with microfluidics, mass spectrometry, or optical absorption spectroscopy may allow the real-time analysis of complex reaction environments with improved chemical specificity. Third, the integration of SERS with advanced nanophotonic platforms may further enhance detection sensitivity and enable new excitation schemes. Recent developments in nanophotonics, including plasmonic nanoantenna [360], photonic crystal cavities [361], and nano-scale quantum light sources, such as single-photon emitters or quantum dots [362], can generate highly confined electromagnetic fields and tailored photon statistics. These devices may enable near-field excitation, quantum-enhanced Raman scattering, and real-time monitoring of molecular interactions at extremely low photon flux. The development of such hybrid SERS–nanophotonic systems could potentially enable super-resolution imaging, ultrafast temporal probing, and highly sensitive molecular detection simultaneously. Fourth, current progress in electronic sensing platforms, particularly wrinkled or crumpled graphene field-effect transistors (FETs), have shown comparable sensitivity for selected biomolecular applications. In these systems, strain-engineered graphene architectures increase effective surface area, improve analyte–surface interactions, and enhance charge-modulated electrical responses, enabling trace-level biomarker detection in complex samples. Combined with signal amplification and data-driven analysis, graphene FET sensors provide rapid, real-time, and highly sensitive electrical readouts. However, their signals mainly rely on changes in electrical properties, which can be influenced by nonspecific adsorption, ionic strength, and matrix interference in complex biological or environmental samples. In addition, electrical readouts generally provide limited intrinsic chemical specificity compared with vibrational spectroscopic techniques. In contrast, SERS provides molecule-specific Raman fingerprint information, allowing sensitive detection together with molecular identification, spectral interpretation, and multiplexed analysis. Therefore, graphene FET sensors and SERS should be viewed as complementary next-generation sensing platforms: graphene FETs are attractive for rapid real-time electrical detection, whereas SERS remains especially powerful for applications requiring simultaneous ultrasensitivity, molecular specificity, and chemical information in clinical diagnostics and environmental monitoring [363,364]. Collectively, these emerging directions are expected to deepen our fundamental understanding of SERS mechanisms while accelerating the development of next-generation analytical technologies for applications ranging from catalysis and advanced materials to biomedical diagnostics.

### 6.2. Nanoparticles and Biomedical Applications

Although there is the rapid development of laser-synthesized NPs, several technological challenges must be addressed to enable their broader biomedical implementation. One of the key limitations remains the scalability of the PLAL method, since conventional laboratory systems often provide relatively low NP yields. Future progress is therefore expected from the use of high-repetition-rate laser sources, burst-mode irradiation, and parallelized ablation geometries such as diffractive optical elements (DOEs), which are capable of significantly increasing production rates. In addition, systematic optimization strategies could enable the rational control of nanoparticle size distribution, concentration, and surface chemistry by simultaneously tuning laser parameters, target composition, and the liquid environment. Emerging artificial-intelligence-assisted optimization of laser synthesis may further accelerate this process through data-driven prediction of nanoparticle properties. Another promising direction involves the fabrication of multi-component and hybrid nanostructures, with well-defined composition and architecture enabling the integration of plasmonic, semiconductor, and catalytic functionalities within a single nanoplatform.

From the biomedical perspective, further progress will strongly depend on the ability to engineer NP surfaces and interfaces in a controlled manner. In situ surface functionalization during laser synthesis, including the incorporation of stabilizing ligands, biomolecules, or polymer coatings directly in the liquid phase, may significantly improve colloidal stability and targeted interaction with biological systems. The integration of PLAL with microfluidic platforms also represents a promising strategy, providing improved control over NP formation and enabling continuous-flow production. In addition, in situ spectroscopic monitoring of laser ablation and nanoparticle growth can provide valuable insight into formation mechanisms, thereby allowing more precise control of nanoparticle size, structure, and composition. Such advances could expand biomedical applications of laser-fabricated nanoparticles, including bioimaging, photothermal therapy, antibacterial treatments, and emerging areas such as wound and burn healing, where plasmonic and semiconductor nanostructures are being explored for accelerated tissue regeneration and infection control. Continued progress in scalable laser nanomanufacturing, compositional control of complex nanostructures, and precise surface engineering will therefore be essential for translating PLAL-derived NPs from laboratory studies toward practical biomedical technologies with controlled functionality and improved biocompatibility.

Overall, these advances are expected to position laser-fabricated nanoparticles as versatile building blocks for next-generation, multifunctional nanomedicine platforms combining diagnostic and therapeutic capabilities.

### 6.3. Perspectives of HEA-Based SERS

HEA-SERS nanoprobes are finding use across diagnostics, environmental monitoring, and nuclear safeguards [365]. In point-of-care settings, HEA-NPs have been incorporated into lateral flow assays for glucose and metabolic biomarkers [137,259], while their lower photothermal conversion reduces laser-induced tissue damage for in vivo use [366]. HEA aerogels have been applied to monitor uranyl ions in nitric acid solutions [367], and the self-passivating oxide layer maintains sensor performance in contaminated water samples [132]. Looking ahead, replacing noble metals with earth-abundant transition metals (Fe, Co, Ni, Cu, Mn) could lower the cost barrier to broad adoption [131,368], and high-throughput screening methods are being developed to survey alloy compositions for optimal SERS performance [265].

### 6.4. Challenges in Modeling

Despite impressive progress in modeling and ML, challenges remain. Model generalization across instruments and conditions is difficult: SERS data vary with substrate, spectrometer, and sample preparation. A model trained in one lab may not transfer to another if laser wavelength or spectrometer resolution differs [294,300].

Future solutions include robust feature extraction focusing on relative peak patterns rather than absolute positions, and domain adaptation techniques in ML to adjust models to new conditions [294,300]. Recent implementations of self-attention mechanisms on porous Ag foams demonstrate the potential for pretreatment-free sensing in microplastics [369]. Furthermore, as the field moves toward commercialization, adopting standardized protocols for quantitative analytical SERS remains a priority [370]. As noted in Section 4.2.1, data scarcity for biomedical analytes remains a key bottleneck; the transfer learning and synthetic augmentation strategies already described will play increasingly important roles [294,300].

Multi-modal data integration is an exciting frontier: combining SERS data with other sensor data (fluorescence, mass spec, patient metadata) using AI to improve overall diagnostic accuracy [300]. Physics-informed AI, where ML models are constrained by known physical laws or DFT-derived rules, is emerging as a path to prevent unphysical predictions [58].

Looking forward, foundation models trained on millions of SERS spectra across chemistry space could serve as starting points for specialized applications. SERS is poised to become an increasingly versatile platform for decoding unknown molecular structures and analyzing complicated multi-component systems with high sensitivity and selectivity [10,294,300].

### 6.5. Field Deployability of SERS

Despite showing ultrahigh sensitivity, the field deployment of SERS is restricted by substrate repeatability, environmental fouling, and signal variability. To overcome these challenges, flexible SERS swabs modified with AgNP-PATS and Ag-MOFs have been created, allowing the simultaneous identification of CWAs (DMMP, 2-CEES, and acetonitrile) [371] and explosives (RDX and TNT) [372], respectively, with LODs of 1.0–57.9 µg/L. When combined with smartphone-based signal processing, these solutions dramatically increase on-site quantification and user accessibility. Future research should integrate AI and machine learning with SERS to enhance pattern recognition and minimize false positives in complex environments. While current CWA detection remains limited [373,374,375,376], AI-driven signal processing will provide the intelligence and adaptability required for real-world dynamic sensing [377].

## 7. Conclusions

Recent advances in SERS demonstrate that precise engineering of plasmonic hotspots from hybrid nanostructures enables (i) decreasing detection limits down to the single-molecule level and (ii) increasing enhancement factors up to ~10^6^–10^10^. In particular, multi-component nanostructures integrating plasmonic metals with semiconductors and other functional materials provide synergistic electromagnetic and chemical enhancement, improving both sensitivity and signal reproducibility in complex environments. Laser-fabricated nanoparticles produced by pulsed laser ablation in liquids offer a unique, surfactant-free platform for the synthesis of such structures with controlled composition and surfactant-free surfaces, which are crucial for reliable SERS performance.

Laser processing, especially ultrafast laser processing, is becoming a reliable method to fabricate large-area, high-performance SERS substrates with relatively low cost and time savings. It is expected that ultrafast laser processing will realize sub-5 nm nanostructures on the target surface in the near future, which will produce more attractive SERS substrates that confine the hotspots at specific, accurate positions and achieve single-molecule sensing on a single nanoparticle. In addition, assisted by an AI algorithm, SERS can be used for the analysis of multi-analyte in raw samples, which greatly expands the applications of SERS in real life.

While electromagnetic enhancement is foundational, practical SERS performance depends equally on the interplay between hotspot architecture, surface chemistry, and analyte transport. Recent innovations in precision nanofabrication, interfacial engineering, and active enrichment have moved SERS from controlled laboratory settings toward robust real-world applications. Specifically, advancements in substrate design and flexible sampling have facilitated the trace detection of explosives, chemical warfare agents, and illicit substances. Nevertheless, widespread field deployment remains hampered by issues with reproducibility, environmental stability, and matrix interference. Overcoming these hurdles will require the continued integration of durable substrates, selective molecular interfaces, and data-driven analysis to establish SERS as a definitive tool for forensic and security sensing.

Computation and modeling highlight the synergistic integration of first-principles modeling and machine learning for advancing SERS as a quantitative and predictive analytical tool. Density functional theory provides microscopic insight into molecule–surface interactions and chemical enhancement mechanisms, while machine learning enables robust, high-throughput analysis of complex spectral data. Quantitative benchmarks, including vibrational frequency accuracy, classification performance, and computational speedup, are identified to bridge theoretical predictions with experimental observables. The discussion further demonstrates how integrated DFT–ML workflows enable both data-driven analyte identification and accelerated spectral prediction, significantly enhancing scalability and reliability.

Beyond sensing, SERS also shows strong potential in biomedical applications, including biosensing, clinical diagnostics, and bioimaging, supporting its use in multifunctional nanoplatforms. Overall, this framework establishes a pathway toward real-time, high-accuracy SERS analysis across environmental, biomedical, industrial, and national security applications.

## Figures and Tables

**Table 1:** Four core effects of high entropy alloys and their relevance to SERS.

Effect	Physical Description	Relevance to SERS
High Entropy	Stabilization of single-phase solid solutions over complex intermetallics.	Ensures structural homogeneity and prevents phase-dependent spectral artifacts.
Lattice Distortion	Atomic size mismatches between diverse elements create local strain fields.	Modulates electronic band structures and increases the density of surface defects/active sites.
Sluggish Diffusion	High potential barriers for atomic migration at elevated temperatures.	Improves thermal stability of nanostructures, preventing the collapse of hotspots.
Cocktail Effect	Cooperative combination of individual elemental properties yielding non-additive behavior.	Permits multi-elemental tuning of LSPR and d-band centers for particular molecular targets.

**Table 2:** Mapping between distinctive statistical properties of SERS spectra and the algorithmic inductive biases best matched to them. The table motivates the method-specific theoretical treatments that follow in this subsection.

SERS Data Property	Algorithmic Inductive Bias
High-dimensional, strongly collinear wavenumber channels	Linear projection (PCA, PLS); ℓ_1_/ℓ_2_-regularised regression
Informative content concentrated in a sparse subset of bands	Tree ensembles with feature importance (RF, gradient-boosted trees); sparse linear models
Vibrational peaks are local 1-D patterns	Convolutional filters (1-D CNN); wavelet bases
Modest peak shifts from chemical or substrate variation	Translation-equivariant convolutions; data augmentation in *ω*
Long-range peak correlations (overtones, combination bands)	Self-attention/transformers; deep CNNs with large receptive fields
Strong fluorescence backgrounds and shot noise	Autoencoders; denoising autoencoders; physically-motivated baseline priors
Small labelled datasets (clinical, forensic)	Kernel SVM; Gaussian processes; Bayesian neural networks; transfer learning
Need for calibrated confidence (≥95% deployment thresholds)	Bayesian methods; deep ensembles; conformal prediction
Need for physically realistic data augmentation	GANs; VAEs; DFT-as-prior generative pipelines

**Table 3:** Comparison of representative machine-learning approaches for Raman/SERS spectral analysis. The third column makes explicit the inductive-bias-to-data-property correspondence summarized in Table 1, addressing why each method is suited (or unsuited) to specific SERS deployment regimes. Citations for individual rows follow the numbering of the original Section 4.4 table and are omitted here for compactness.

Approach	Mathematical Core	Inductive Bias/SERS Rationale	Performance & Data	Interpretability	SERS Use Case
***Supervised***					
SVM (RBF kernel)	*min* ½‖w‖^2^ + C∑ξ; kernel *k*(*x*,*x*′)	Kernel similarity tolerates scarce data; no locality bias	≈90%, *N* = 50–200	Moderate	Bacteria classification
Random Forest	Ensemble of decorrelated trees; threshold splits	Feature importances localise discriminative bands; baseline-robust	≈90%, *N* = 50–200	High	Explosives detection
Gradient boosting	Boosted decision trees (XGBoost/LightGBM)	Stronger fitting on tabular spectral features than RF; preserves interpretability	92–95%, *N* = 50–500	Moderate–High	Quantitative classification
PLS Regression	Latent components *t_i_* maximising cov(*t*, *y*)	Handles channel collinearity; loadings interpretable as virtual spectra	Good, *N* = 30–100	High	Concentration regression
***Unsupervised***					
PCA/LDA	Eigendecomposition of covariance	Linear denoising via dominant variance modes; fails under non-linear peak shifts	Good	High	Clustering, preprocessing
t-SNE/UMAP	KL/cross-entropy on neighbour probabilities	Non-linear manifolds absorb peak shifts; preserve local structure	Excellent visualisation	Moderate	Visualisation; biological subtypes
K-means/GMM	Centroid/likelihood partitioning	Anomaly detection via density modelling of normal spectra	Moderate	Moderate	Grouping; QC anomaly flagging
***Deep learning***					
1-D CNN	Local equivariant convolutions	Locality + shift-equivariance match local, shift-prone vibrational bands	≥95%, *N* > 10^3^	Moderate (CAM/Grad-CAM)	Virus/pathogen detection
1-D Transformer	Self-attention over spectral channels	Captures long-range peak correlations (overtones, combination bands)	≥95%, *N* > 10^3^–10^4^	Moderate (attention maps)	Multiplexed identification
Autoencoder/VAE	Bottleneck reconstruction; KL-regularised latent	Learned non-linear baseline removal; latent likelihood for novelty	Good, *N* > 10^3^	Moderate	Denoising; novelty detection
GAN	Adversarial min–max	Synthetic augmentation for clinical data scarcity; mode-collapse risk	Good, *N* > 10^3^	Low	Data augmentation
GNN (Mol2Raman)	Message passing on molecular graph	Atomic permutation/bond locality match vibrational normal modes	High accuracy	Moderate	Inverse design; DFT surrogate
***Probabilistic/hybrid***					
Gaussian process	Posterior over functions	Calibrated uncertainty for low-data and safety-critical decisions	Good, *N* < 200	High	Uncertainty-aware sensing
Bayesian NN/deep ensemble	Approximate posterior or ensemble averaging	Calibrated uncertainty in deep-learning regime	Good, *N* > 10^3^	Moderate	Clinical decision support
DFT + ML hybrid	Physics-grounded data augmentation	Principled training data; complements adversarial augmentation	Excellent, moderate N	High	Molecule ID for unseen analytes
Transfer learning	*L_T_* + λ Ω(θ − θ*_S_*)	Low-level features instrument-invariant; high-level task-specific	Excellent, small target N	Moderate	Cross-instrument adaptation

**Table 4:** SERS-based detection of explosives, CWAs, and illicit drugs: substrates and sensing strategies.

Target Analyte	SERS Substrate/Material	Limit of Detection	Key Strategy/Feature	Reference
NTO, FOX-7, TNT	Ag-ZnO nanoflower substrate	10^−6^–10^−14^ M	Electromagnetic hotspot enhancement	[334]
PA, RDX	Ag-Au nanoparticle micro square array	3.6 × 10^−8^ M (PA), 4 × 10^−7^ M (RDX)	Mobile nanomotors enable real time SERS detection	[350]
CL-20, TNCB	Alkali ion assisted SERS on Ag surface	Sub-nanomolar	Electrostatic complex formation	[336]
NTO, TNB, PA	TiO_2_ aerogel semiconductor	2.42 × 10^−7^ M	Oxygen vacancies enhance charge transfer Raman enhancement	[337]
DMMP (sarin simulant)	Ag nanoplatelets on graphite	2.5 ppm	Gas phase SERS detection using portable Raman device	[341]
VX nerve agent	AuNP decorated quartz fibres	0.008 µg/L	Fiber based plasmonic substrate enables vapor detection	[343]
Nito explosives, drugs	NH_2_-MIL-101(Fe)@AuNP core shell substrate, MIL-1-1(Cr), ZIF-8	0.46–2.31 ng/mL	MOF assisted adsorption enhances sensitivity	[309,351,352]
VX, Tabun, Cyclosarin	Au coated nanopillar substrate with oxime capture molecules	Sub-ppm	Functionalized biorecognition interface improves selectivity	[353]
Fentanyl (serum/urine)	Liquid–liquid interfacial AuNP plasmonic arrays	1 ng/mL	Direct detection without pretreatment	[309]
	Au nanostar decorated rGO	~0.47 ng/mL	Hybrid plasmonic graphene substrate	[354]
Tramadol (serum)	Au trisoctahedra on flexible tape	69.19 ng/mL	Wearable sensing platform for forensic detection	[347]
Methamphetamine (wastewater)	Au@Ag modified fibrous paper substrate	Sub-ppb	Paper-based flexible SERS sensor	[349]

## Data Availability

No new data were created in this work.
